# Resolvin D1 reduces cancer growth stimulating a protective neutrophil-dependent recruitment of anti-tumor monocytes

**DOI:** 10.1186/s13046-021-01937-3

**Published:** 2021-04-12

**Authors:** Domenico Mattoscio, Elisa Isopi, Alessia Lamolinara, Sara Patruno, Alessandro Medda, Federica De Cecco, Susanna Chiocca, Manuela Iezzi, Mario Romano, Antonio Recchiuti

**Affiliations:** 1 Department of Medical, Oral, and Biotechnology Science, University “G. d’Annunzio” Chieti-Pescara, Chieti, Italy; 2 Center for Advanced Studies and Technology (CAST), University “G. d’Annunzio” Chieti-Pescara, Chieti, Italy; 3 Department of Neuroscience, Imaging and Clinical Sciences, University “G. d’Annunzio” Chieti-Pescara, Chieti, Italy; 4 Department of Experimental Oncology, IEO, European Institute of Oncology IRCCS, Milan, Italy

**Keywords:** Resolvin D1, HPV cancers, Resolution of inflammation, Neutrophils, Classical monocytes

## Abstract

**Background:**

Innovative therapies to target tumor-associated neutrophils (PMN) are of clinical interest, since these cells are centrally involved in cancer inflammation and tumor progression. Resolvin D1 (RvD1) is a lipid autacoid that promotes resolution of inflammation by regulating the activity of distinct immune and non-immune cells. Here, using human papilloma virus (HPV) tumorigenesis as a model, we investigated whether RvD1 modulates PMN to reduce tumor progression.

**Methods:**

Growth-curve assays with multiple cell lines and in vivo grafting of two distinct HPV-positive cells in syngeneic mice were used to determine if RvD1 reduced cancer growth. To investigate if and how RvD1 modulates PMN activities, RNA sequencing and multiplex cytokine ELISA of human PMN in co-culture with HPV-positive cells, coupled with pharmacological depletion of PMN in vivo, were performed. The mouse intratumoral immune cell composition was evaluated through FACS analysis. Growth-curve assays and in vivo pharmacological depletion were used to evaluate anti-tumor activities of human and mouse monocytes, respectively. Bioinformatic analysis of The Cancer Genome Atlas (TCGA) database was exploited to validate experimental findings in patients.

**Results:**

RvD1 decreased in vitro and in vivo proliferation of human and mouse HPV-positive cancer cells through stimulation of PMN anti-tumor activities. In addition, RvD1 stimulated a PMN-dependent recruitment of classical monocytes as key determinant to reduce tumor growth in vivo. In human in vitro systems, exposure of PMN to RvD1 increased the production of the monocyte chemoattractant protein-1 (MCP-1), and enhanced transmigration of classical monocytes, with potent anti-tumor actions, toward HPV-positive cancer cells. Consistently, mining of immune cells infiltration levels in cervical cancer patients from the TCGA database evidenced an enhanced immune reaction and better clinical outcomes in patients with higher intratumoral monocytes as compared to patients with higher PMN infiltration.

**Conclusions:**

RvD1 reduces cancer growth by activating PMN anti-cancer activities and encouraging a protective PMN-dependent recruitment of anti-tumor monocytes. These findings demonstrate efficacy of RvD1 as an innovative therapeutic able to stimulate PMN reprogramming to an anti-cancer phenotype that restrains tumor growth.

**Supplementary Information:**

The online version contains supplementary material available at 10.1186/s13046-021-01937-3.

## Background

A substantial body of evidence demonstrates that chronic, non-resolving inflammation sustains the development and progression of several tumors [1, 2]. The unbalanced production of inflammatory mediators and the presence and activation of an inflammatory infiltrate in the cancer microenvironment may be hijacked by tumors to foster their proliferation, survival and migration. Among inflammatory cells, polymorphonuclear neutrophils (PMN) are recognized as relevant drivers of inflammation and tumorigenesis [3, 4]. Indeed, PMN are the first leukocytes to be rapidly recruited at sites of acute inflammation to remove the damaging agent and to launch a wave of monocyte infiltration [5]. PMN subsequently undergo apoptosis and are cleared by monocyte-differentiated macrophages (MΦs) through efferocytosis [6]. Although PMN recruitment is a crucial host defense response, an excessive and uncontrolled PMN infiltration may promote chronic inflammation and tissue damage [7]. Along these lines, cancer progression can be considered as a chronic inflammatory reaction, characterized by persistent PMN infiltration in the tumor microenvironment [8]. PMN drive chronic inflammation and sustain oncogenesis by releasing inflammatory mediators, promoting angiogenesis and invasion, and blunting the innate and adaptive immunity against cancer cells [4] (pro-tumorigenic or N2-polarized PMN [9]). Consistent with their cancerogenic role, elevated PMN infiltration correlates with a poor prognosis in several cancers [10] (recently reviewed in [11]). On the other hand, PMN can also exert potent anti-tumor functions (anti-tumorigenic or N1-PMN [9]), such as cytotoxicity and/or recruitment of protective inflammatory cells, suggesting the possibility to manipulate PMN for anti-cancer purposes [12]. Therefore, approaches aimed at PMN re-education during tumorigenesis are of interest.

Resolution of inflammation is an active process characterized by the reduction of PMN infiltration in inflammatory sites. This process is governed by a switch in the endogenous production of lipid autacoids from pro-inflammatory to pro-resolving, collectively termed specialized pro-resolving lipid mediators (SPMs). Resolvins (Rv), encompassing RvD1–6, are a class of SPMs that regulate PMN infiltration and activation, enhance MΦs efferocytosis, stimulate the active removal of pathogens and control the balance of pro- and anti-inflammatory cytokine production [13]. Due to their potent bioactions, Rv proved beneficial in experimental diseases characterized by unrelenting inflammation (reviewed in [14]). Rv and their aspirin-triggered metabolites also potentiate NK cytotoxic activity against adenocarcinoma cells [15], promote the removal of therapy-generated tumor cell debris [16, 17], reduce neoplasm growth in oral squamous cell carcinoma [18], and eradicate micro metastasis when preoperative administered [19], suggesting that the restoration of an appropriate resolution program may prove beneficial also in cancers. However, the mechanisms of the emerging Rv anti-tumors activity are still incompletely known. Here, we show that in a human papilloma virus (HPV) model of tumorigenesis, RvD1 reprograms the PMN and stimulates an intra-tumoral recruitment of anti-cancer monocytes that inhibit tumor growth.

## Methods

### Cells

HeLa (ATCC), A549 (ATCC) and UM-SCC-104 cells (Merck KGaA) were maintained in DMEM (Merck KGaA) plus 10% FBS (Life Technologies). C3 and TC-1 were kindly provided by Dr. A. Venuti (IRCCS Regina Elena National Cancer Institute, Italy) and maintained in RPMI (Merck KGaA) plus 10% FBS. C3 and TC-1 were grown and amplified in vitro before allograft.

### PMN and monocyte purification

Buffy-coats from healthy donors were obtained from the ASL2 Lanciano-Vasto-Chieti (Abruzzo, Italy) transfusion center after informed consent. Blood-derived PMN and monocytes were isolated by density-gradient centrifugation. Monocytes were then purified from the peripheral blood mononuclear cells (PBMCs) by adhesion on tissue culture dishes. Briefly, PBMCs were seeded in RPMI without serum for 30 min at 37 °C, and non-adherent cells (lymphocytes) were removed. Adherent cells (monocytes) were washed, collected by trypsinization and resuspended in RPMI+ 10% serum for experiments. PMN were recovered after hypotonic lysis of red blood cells with ultra-pure water (Millipore) for 30 s at room temperature and maintained in RPMI+ 10% FBS at 10 × 10^6^ cells/ml during experiments.

Classical monocytes were purified from human PBMCs by positive selection using CD14-conjugated magnetic beads (Miltenyi Biotec) according to the manufacturer’s instructions. Purity of cell populations was evaluated by flow cytometry and exceeded 95%. Monocytes were resuspended and maintained in RPMI+ 10% FBS during experiments.

Mouse PMN were isolated from bone marrow of C57BL/6 mice with a Hystopaque (Merk KGaA)-based density gradient centrifugation and maintained in RPMI+ 10% FBS during experiments.

### In vitro cell growth analysis

For cell growth studies, 10 × 10^4^ HeLa, UM-SCC-104, A549, or C3 cells (target cells) were seeded on 12-well plates 2 h before treatments and PMN addition (effector cells). Freshly isolated blood-derived human PMN were exposed to vehicle (0.5% vol/vol EtOH in PBS) or 100 nM RvD1 (Cayman Chemicals) for 15 min at 37 °C and then added on top of cancer cell lines at an effector: target (E:T) ratio of 10:1 (HeLa and A549) or 30:1 (UM-SCC-104). Freshly isolated bone marrow-derived mouse PMN were treated with RvD1 as described above and added on top of C3 cells at an E:T ratio of 10:1. Human or mouse cancer cells exposed to vehicle or RvD1 but without PMN were used as control. Eighteen hours after co-incubations, adherent cancer cells were enumerated by crystal violet staining.

For the impedance-based real time cell analysis, 5 × 10^4^ HeLa or C3 cells were seeded on iCelligence E-plate L8 (ACEA Biosciences) and treated with RvD1 0–1–10-100 nM. Cell growth was monitored continuously up to 65 h with the ACEA instrument. To analyze the impact of PMN on HeLa cell growth, 5 × 10^4^ HeLa cells were seeded on iCelligence E-plate L8 2 h before PMN addition. Freshly isolated blood-derived PMN were treated with RvD1 100 nM for 15 min at 37 °C and then added on top of HeLa cells at an E:T ratio of 10:1. Cell growth was monitored continuously up to 20 h after PMN addition. To study anticancer activity of monocytes, purified CD14^+^ monocytes were added on top of HeLa seeded on iCelligence E-plate L8 at an E:T ratio of 10:1. Cell growth was monitored up to 72 h.

### Apoptosis and NETtosis assays

Blood-derived human PMN were treated with vehicle or RvD1 100 nM as described above and added on top of HeLa cells at an E:T ratio of 10:1. After 18 h of co-incubation, PMN were harvested, counted and stained for FACS analysis, and supernatants were collected for western blotting of citrullinated histone H3 and for quantification of extracellular DNA release with the Qubit fluorometer (Life technologies). For apoptosis, 5 × 10^5^ cells were incubated for 15 min at room temperature with Annexin V-FITC (Merck KGaA), washed, incubated with 50 μg/ml Propidium Iodide (PI) and immediately processed. Samples were acquired using a FACSCanto II flow cytometer (Becton Dickinson), and data were analyzed with the FACS Diva software (BD Bioscience). For evaluation of neutrophil extracellular traps (NETs) formation, supernatants from an equal number of PMN (1 × 10^6^) were resolved by SDS-PAGE under reducing conditions and immunoblotted with the anti-citrullinated histone H3 (Abcam) as marker of NETtosis.

### RNAseq and bioinformatic analysis

Blood-derived human PMN were treated with vehicle or RvD1 100 nM as described above and added on top of HeLa cells at an E:T ratio of 10:1. Treated PMN without HeLa cells were used as control. After 3 h of co-incubation, PMN were harvested, washed, lysed with Trizol (ThermoFisher Scientific), and RNA was extracted from cells with the Quick-RNA MiniPrep kit according to the manufacturer’s instructions (Zymo Research). RNA quality and integrity were assessed with an Agilent 2100 bioanalyzer using an RNA 6000 Nano Kit (Agilent Technologies). mRNA-Seq library was prepared from 500 ng of total RNA with the TruSeq Stranded Total RNA (Illumina) and sequenced with Novaseq 6000 (Illumina) with a read length of 50 bp (paired-end). FastX-toolkits and FastQC tools were used to filter reads and control the quality of all raw data; TopHat was used for the alignment of reads to the reference genome. Bioconductor tools EdgeR and DESeq2 were used to calculate absolute and differential gene expression.

The Ingenuity Pathway Analysis (IPA) (Qiagen) tool was used to evaluate diseases and functions affected by RvD1 in PMN. IPA was carried out using a ± 1.5-fold cut-off to identify differentially expressed genes, including direct and indirect relationships in human neutrophils with an experimentally observed or high (predicted) confidence. *P* < 0.05 identified significantly affected pathways and functions, with positive or negative z-score values indicating activation or inhibition, respectively.

### Monocyte chemotaxis

Freshly isolated blood-derived human PMN were treated with vehicle or RvD1 100 nM and added on top of HeLa cells seeded in the bottom chamber of a Transwell (Corning) insert at an E:T ratio of 10:1. Monocytes were added in the upper chamber of the Transwell at 1:1 ratio with PMN, separated from the bottom compartment by a 3 μM-pore filter, and incubated for 18 h. At the end of the experiments, migrated monocytes were collected from the bottom side of the filter and the bottom chamber, stained with anti-CD14 and CD16 antibodies (Biolegend) and analyzed by flow cytometry. Relative monocyte migration was determined by counting the number of CD14^+^CD16^−^ and CD14^low^CD16^+^ events recorded in 30 s of acquisition.

### Multiplex ELISA

Eleven cytokines and chemokines were quantified in cell culture supernatants using a MILLIPLEX MAP Kit (HCYTOMAG-60 K, Millipore). Briefly, healthy human PMN were cocultured with HeLa cells for 18 h in the presence of vehicle or RvD1 (100 nM). At the end of co-incubations, cell-free supernatants were used to measure cytokine/chemokine concentration on a Luminex 200 platform (Luminex Corporation) following manufacturers’ instructions. Data were analyzed with the xPONENT 3.1 software. Cytokine/chemokine concentrations in the samples were determined with a 5-parameter logistic curve. Final concentrations were calculated from the mean fluorescence intensity and expressed as pg/mL.

### Animal studies

Six/eight weeks-old C57BL/6 male and female mice were purchased from Charles River Laboratories and housed under specific pathogen-free conditions (T = 25 °C, 12 h/12 h light/dark cycle) with unrestricted access to food and water.

C3 (50 × 10^4^) or TC-1 (5 × 10^4^) TC-1 cells were injected subcutaneously in the right flank of mice in a volume of 200 μl PBS. When tumors became palpable, mice were randomized and treated with vehicle (0.5% vol/vol EtOH in NaCl 0.9%) or RvD1 at 100 ng/mouse, by intra peritoneal (i.p.) administration in the lower right abdominal quadrant three times per week.

For PMN depletion, mice were treated with 200 μg of anti-IgG as isotype control or anti-Ly6G (clone 1A8, BioXCell) respectively, by i.p. administration every other day starting at tumor gross appearance, for a total of six doses in 12 days of treatment. Vehicle or RvD1 were simultaneously administered to generate four groups of mice: vehicle+IgG, vehicle+Ly6G, RvD1 + IgG, RvD1 + Ly6G.

To dampen classical monocyte recruitment in tumors, tumor bearing mice were treated with the CCR2 antagonist SC-202525 (Santa Cruz Biotechnology) that was administered i.p. at 2 mg /kg (in saline) per mouse as reported [20, 21], three times per week starting at the gross appearance of tumors. Vehicle or RvD1 were simultaneously administered to generate four groups of mice: vehicle (0.5% + 0.5% vol/vol EtOH in 0.9%NaCl), RvD1 (100 ng/mouse+ 0.5 vol/vol EtOH), CCR2 antagonist (0.5% vol/vol EtOH+SC-202525), CCR2 inhib+RvD1 (SC-202525 + RvD1).

Tumor growth was measured biweekly with a caliper and reported accordingly to the formula: V = (width)^2^ x length/2. Mice with tumors> 1500 mm^3^ were euthanized before the termination of the experiment.

Mice were sacrificed 12 days after the initial treatment, unless otherwise specified, by inhalation of carbon dioxide according to the AVMA Guidelines for the Euthanasia of Animals. The tumor was then exposed by vertical cut of the skin and recovered for downstream analysis.

### FACS analysis

Tumors were dissociated with the GentleMACS Dissociator (Miltenyi Biotech) in serum free RPMI plus 100 μg/ml DNAse I (Merck KGaA) and 400 μg/ml collagenase (Merck KGaA), filtered with 70 μm cells strainers (BD Biosciences) and collected by centrifugation. For red blood cell lysis, cell suspensions were incubated with ultra-pure water for 2 min at room temperature, collected by centrifugation and suspended with MACS buffer (Miltenyi Biotech). Single-cell suspensions were then stained (30 min, 4 °C) with 0.2 μg/5 × 10^5^ cells of fluorochrome- tagged antibodies (all from Biolegend) against the following antigens: CD45 (clone 30-F11), CD11b (clone M1/7), Ly6C (clone HK1.4), F4/80 (clone BM8), CD66a (clone Mab-CC1), CD4 (GK1.5), CD8 (53–6.7), CD209 (MMD3). Samples were acquired with a FACS Canto II flow cytometer and analyzed with the FACS Diva software.

### Immunohistochemical and immunofluorescence analyses

Tumor samples were fixed with 10% neutral buffered formalin and embedded into paraffin or fixed with 1% PFA and frozen in a cryo-embedding medium (OCT, Bio-Optica). Slides were cut and stained with Hematoxylin (Bio-Optica) and Eosin (Bio-Optica) for histological examination. Slides were then deparaffinized, serially rehydrated and stained with mouse monoclonal anti-PCNA (Dako), rabbit polyclonal anti-Caspase3 (R&D System), rat monoclonal anti-CD31 (Dianova), followed by the appropriate secondary antibodies. Immunoreactive antigens were detected using streptavidin peroxidase (ThermoScientific) and the DAB Chromogen System (Dako) or alkaline phosphatase-conjugated streptavidin (ThermoScientific) and Vulcan fast red Chromogen (Biocare Medical). After chromogen incubation, slides were counterstained with Hematoxylin (Bio-Optica) and images were acquired by a Leica DMRD optical microscope (Leica). The percentage of PCNA positive cells was determined on digital images of 5–7 tumors per group (2–4 X 200 microscopic fields per sample); clear red nuclei were regarded as positive cells and were selected with the magic wand tool of Photoshop. For each field, the number of positive cell pixels indicated in the histogram window was reported as % on the number of negative cell pixels (PCNA index).

For immunofluorescence evaluation of frozen samples, sections were air-dried, fixed in ice-cold acetone for 10 min and incubated with a Rabbit polyclonal anti-Neutrophil Elastase antibody (Abcam), followed by a secondary antibody conjugated with Alexa 488 (Life Technologies). Image acquisition was performed using a Zeiss LSM 800 confocal microscope.

### Human cancer datasets and analysis

The human cervical squamous cell carcinoma (CESC) RNA-seq data and clinical data from TCGA were retrieved from cBioPortal (https://www.cbioportal.org/datasets). CESC tumor samples were ranked accordingly to PMN, monocytes, CD8 T cells, M1 MΦs infiltration levels as predicted by XCell (https://xcell.ucsf.edu), TIMER2.0 (timer.cistrome.org), or TIP (http://biocc.hrbmu.edu.cn/TIP). Gene set enrichment analysis was carried out with GSEA (http://www.broadinstitute.org/gsea) to determine pathways that showed statistically significant and concordant differences in selected tumor samples, using the GSEA software package and the MSigDB gene sets h (hallmarks), C2:CP (canonical pathways) and C5 (Gene Ontology gene sets). Parameters used for the analysis of TCGA samples: 1000 permutation, phenotype (permutation type), weighted (enrichment statistic), signal2noise (metric for ranking genes). Gene sets significantly enriched with a nominal *P* value < 1% were considered significant.

### Statistical analysis

Statistical significance was evaluated with Graphpad Prism version 7.00 software. Multiple samples were compared with ANOVA. Paired or unpaired Student’s t tests were used to compare two samples normally distributed. One-sample t-test was used to compare two nonparametric populations. Pearson rank correlation coefficient was used to test association between variables. Survival in animal experiments was determined by time-to-event analysis, as previously reported [22]. Survival curves were then estimated by the Kaplan–Meier method and compared using the Mantel–Cox log rank test and the Mantel–Haenszel HR in GraphPad Prism.

Data are expressed as mean ± SEM of the number of biological replicates indicated in each figure legend. Values of *P* < 0.05 were considered significant.

## Results

### RvD1 diminishes tumorigenic growth of transplanted HPV-positive cells

**Fig. 1:**
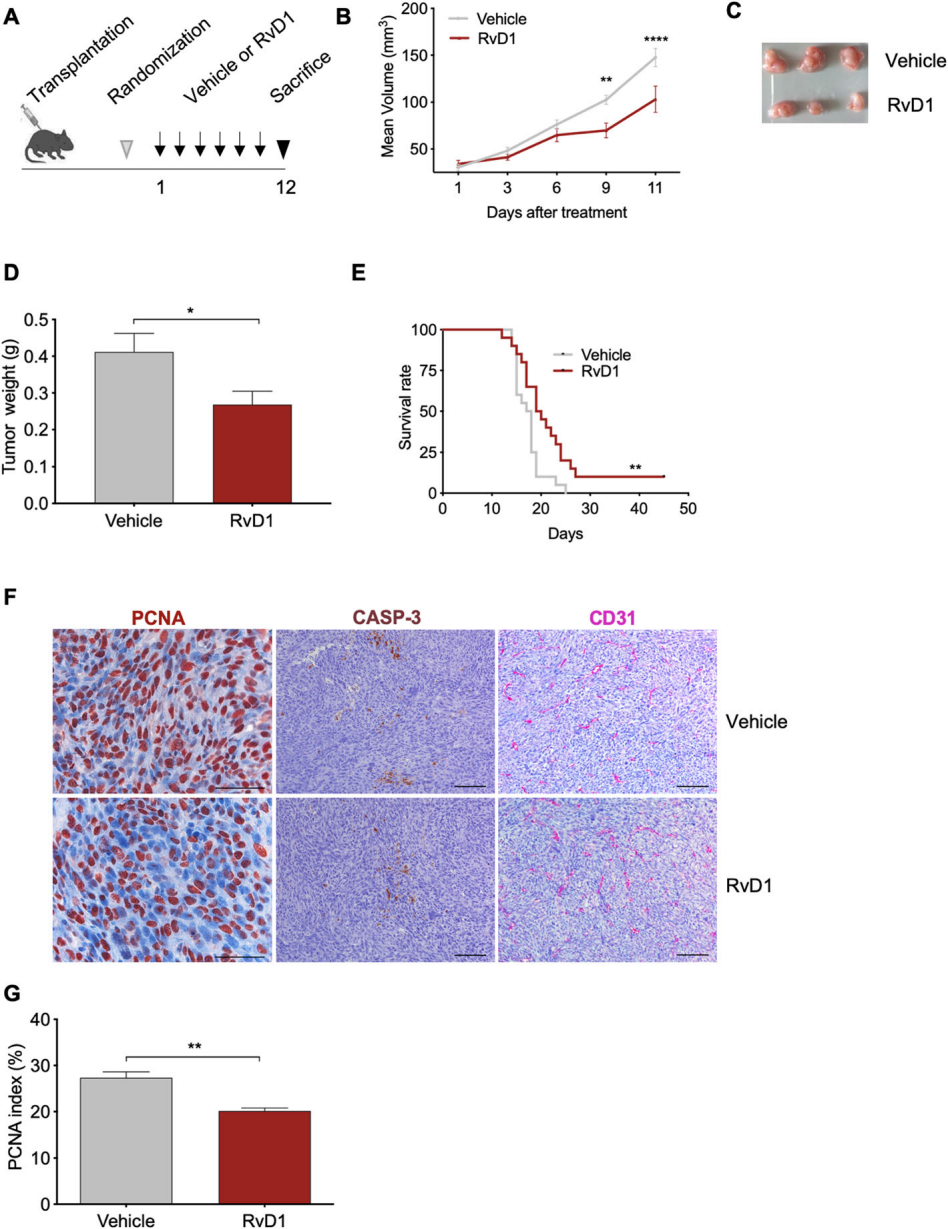
RvD1 reduces tumor growth in syngeneic mice transplanted with HPV-positive C3 cells. **a** Scheme showing in vivo transplantation and treatment protocols, as detailed in Materials and Methods. **b** Mean tumor volume from mice subcutaneously transplanted with C3 cells and treated with Vehicle or RvD1. *n* = 19 mice per group. **, *P* < 0.01; ****, *P* < 0.0001(two-way ANOVA and Sidak’s multiple comparisons test). **c** Representative image showing tumors at sacrifice. **d** Weight of tumors at sacrifice. *n* = 7–10 mice per group *, *P* < 0.05 (Unpaired t test with Welch’s correction). **e** Kaplan-Meier curve survival analysis reporting the time for tumors to reach a volume of 1500 mm^3^ (humanized endpoint). *n* = 18–20 mice per group **, *P* < 0.01 (Log-rank Mantel-Cox test). **f** Representative microsections of tumors biopsies from mice treated with vehicle or RvD1 and stained with antibodies against PCNA (proliferation marker), CASP-3 (apoptotic marker), and CD31 (blood vessels marker). **g** Semiquantitative proliferation score. *n* = 5–7 tumors per group. **, *P* < 0.01

RvD1 anticancer activity was examined in a mouse model of HPV tumorigenesis, formerly used as platform for testing anti-HPV vaccines [23]. HPV-positive C3 cells were subcutaneously transplanted into male and female C57BL/6 mice and treated by intraperitoneal (i.p.) administration of vehicle (0.5% EtOH) or RvD1 (1 μg/kg in 0.9% NaCl) up to 12 days (Fig. 1a). Compared to vehicle, RvD1 reduced tumor volume and weight (Fig. 1b-d) and extended, from 17.5 to 19.5 days (hazard ratio vehicle/RvD1 = 2.712), the mean survival of mice (Fig. 1e), computed as the time for tumors to reach a volume of 1500 mm^3^ (humanized endpoint). Similar effects were denoted when the HPV-positive tumor cell line TC-1 was used in a similar experimental setting (Supplemental material, Fig. S1), thus excluding cell-specific effects. Histological analysis of tumor slides revealed that RvD1 reduced tumor cell proliferation while unaffecting tumor cell apoptosis and blood vessel formation (Fig. 1f-g). However, RvD1 did not decrease proliferation of mouse C3 or human HPV-positive HeLa cells in culture (Supplemental material, Fig. S2), suggesting the involvement of other cells in the RvD1-dependent reduction in tumor proliferation and growth.

### RvD1 inhibition of HPV-tumor development is PMN-dependent

To understand cellular mechanisms of the RvD1 actions, we focused on PMN since their paramount importance in inflammation, resolution and tumorigenesis [3, 4]. Bioinformatic analysis of TCGA transcriptomic data derived from bulk tumor samples of cervical carcinoma patients (CESC), the most frequent cancer associated with HPV infections [24], predicted low PMN infiltration in cervical tumors (Supplemental material, Fig. S3A). However, patients with relatively high PMN infiltration showed shorter overall survival compared to patients with a relatively low number of infiltrated PMN (median months survival: 45.11 vs 134.33, Supplemental material, Fig. S3B), indicating that PMN infiltration dictates cervical cancer outcome, as also reported in experimental data with smaller cohorts of biopsies [25]. Importantly, stratification of CESC tumors accordingly to their TNM stage highlighted that PMN infiltration is higher in T1A-B, thus suggesting a key role for PMN in lower grade of CESC tumors (Supplemental material, Fig. S3C) that deserves further investigations.

In addition, patients with higher inferred CD8 T cells and higher M1 MΦs showed better prognosis (Supplemental material, Fig. S4A-B), as experimentally determined [26, 27], further validating our bioinformatic analysis carried out with the xCell software. Moreover, other software such as TIP, CIBERSORT, MCPCounter, and Quantiseq, confirmed that tumor biopsies with a gene signature predictive of high PMN infiltration are associated with the worst outcome of CESC patients as well as in other cancer types (Supplemental material, Fig. S3D-E), such as adrenocortical carcinoma, glioblastoma multiforme and breast invasive carcinoma. Therefore, targeting PMN in cancer may prove beneficial.

**Fig. 2:**
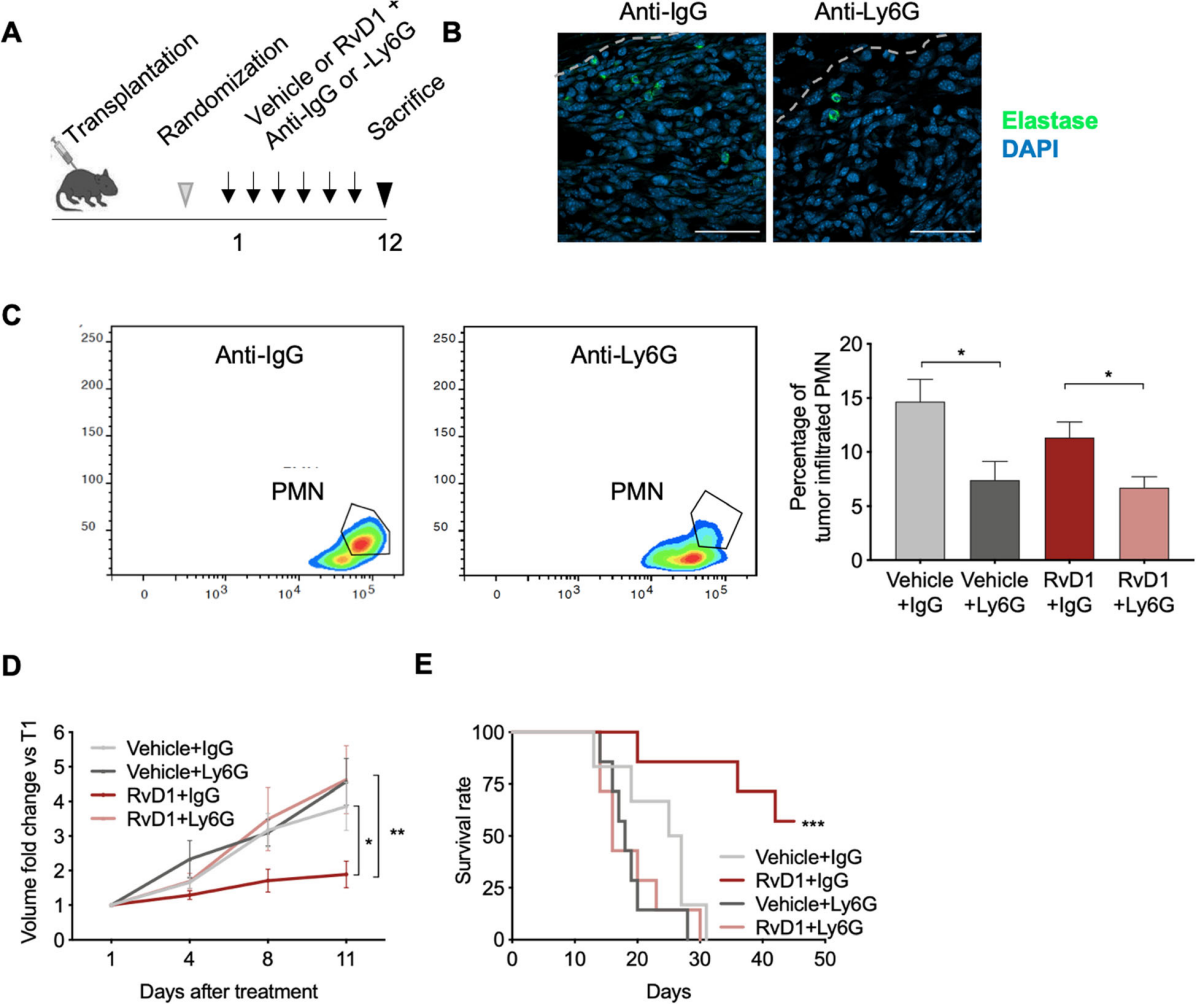
PMN depletion abolishes RvD1 anti-cancer effects. **a** Scheme of the in vivo transplantation of C3 cells in the right flank of C57BL/6 mice, treated with vehicle or RvD1 plus anti-IgG or -Ly6G antibodies three times a week. **b** Representative IF analysis of tumor slides from mice treated with anti-IgG or anti-Ly6G and stained with an anti-elastase antibody to detect tumor-infiltrated PMN. Nuclei were counterstained with DAPI. Tumor edges were marked with dotted lines. Scale bar: 50 μM. **c** Left and middle: Representative flow cytometric counter plot of single-cell dissociated tumors from mice treated with anti-IgG or anti-Ly6G, as indicated, and identified using the strategy reported in Fig. S6. Right: Percentage of tumor-infiltrated PMN determined by FACS analysis of CD66a^+^ cells. Data are expressed as percentage of CD11b^+^ cells. *n* = 4–7 mice per group. *, *P* < 0.05 (two-tailed unpaired *t* test). **d** Tumor growth of C3 cells in mice treated with vehicle/RvD1 plus anti-IgG/−Ly6G antibodies. Data are expressed as volume fold change as compared to the initial tumor volume at the start of treatments (T1). *n* = 5–6 mice per group. * *P* < 0.05 vehicle+IgG vs RvD1 + IgG at day 11 (two-way ANOVA and Tukey’s multiple comparison test); ** *P* < 0.01 RvD1 + IgG vs RvD1 + Ly6G at day 11 (two-way ANOVA and Tukey’s multiple comparison test). **d** Kaplan-Meier curve survival analysis reporting the time for tumors to reach a volume of 1500 mm^3^ (humanized endpoint). *n* = 6–7 mice tumors per group. ***, *P* < 0.001 RvD1 + IgG vs RvD1 + Ly6G (Log-rank Mantel-Cox test)

To test whether RvD1 could modify PMN actions during tumorigenesis, we depleted PMN in C3 cell transplanted C57BL/6 mice by i.p. administration of the selective anti-Ly6G antibody [28] (Fig. 2a). Anti-Ly6G treatment significantly reduced PMN infiltration in both RvD1-treated and -untreated mice, as shown by immunofluorescence images of tumor slides (Fig. 2b) and by FACS analysis of single-cell dissociated tumors) (Fig. 2c). Despite a non-complete depletion of tumor PMN, probably due to an increased PMN production by the bone marrow, production of antibodies against anti-Ly6G by the treated mice, low bioavailability, or induction of extra-medullary granulopoiesis [29], the anti-cancer actions of RvD1 were abolished by the anti-Ly6G antibody (mean volume at day 9 in mm^3^: RvD1 + IgG = 78.31 vs RvD1 + Ly6G = 178, SE of differences 39.33; mean weight at sacrifice: 0.16 g ± 0.02915 vs 0.2483 g ± 0.09569) (Fig. 2d). Moreover, PMN depletion abolished the RvD1-induced extension of mice survival (Fig. 2e). Collectively, these results indicate that PMN play a key role in the RvD1 anti-cancer activity.

### RvD1 modulates PMN activity against cancer cells

**Fig. 3:**
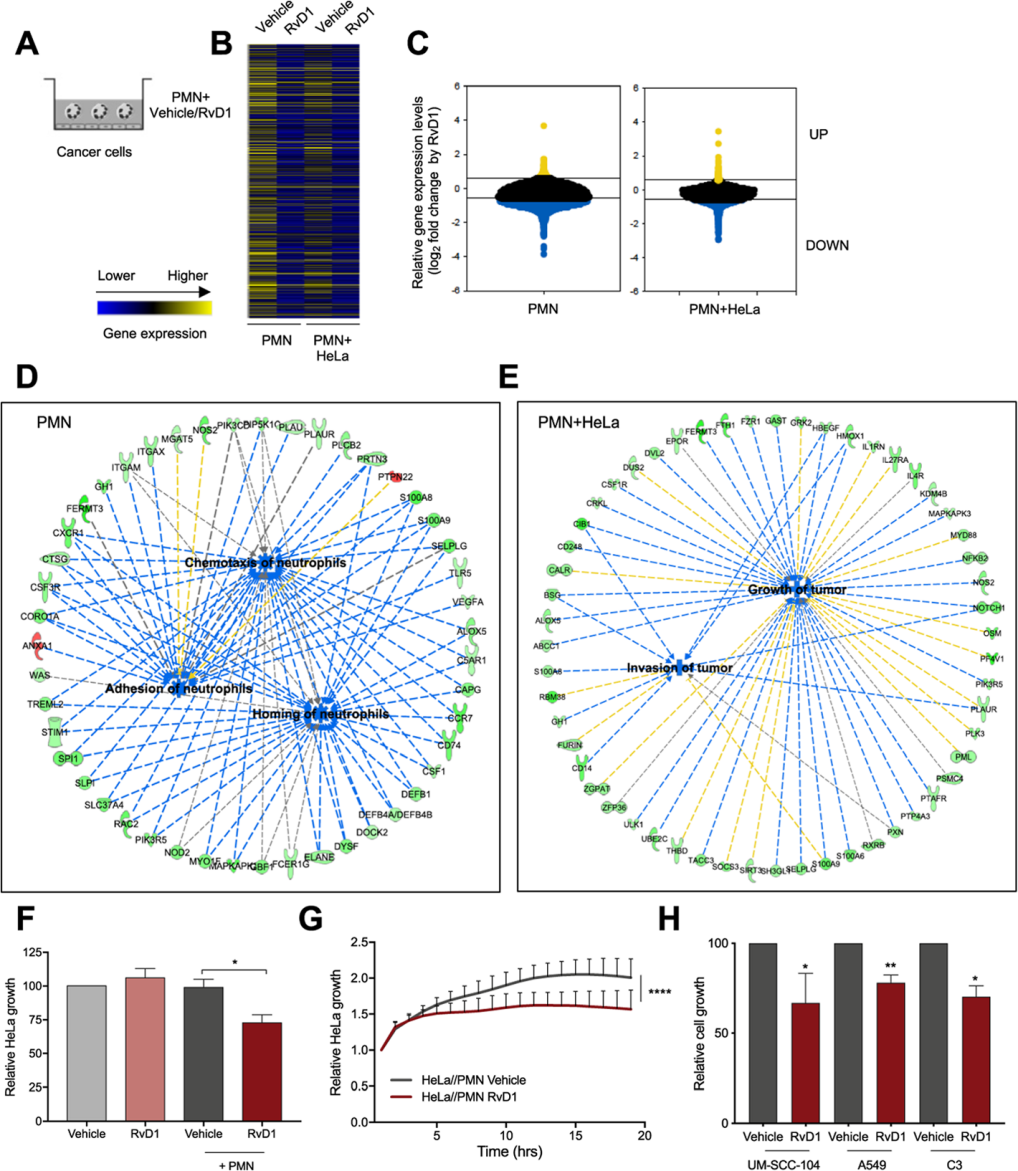
RvD1 regulates PMN gene expression and activity during co-incubation with cancer cell lines. **a** Scheme depicting in vitro PMN/cancer cell co-incubations and treatment. **b** Heatmap view (Morpheus, https://software.broadinstitute.org/morpheus) of gene expression levels (mean of RPKM values of *n* = 4–6 biological replicates pooled in 2–3 samples, respectively) of blood-derived PMN, treated with vehicle or RvD1, alone (PMN) or during co-incubation with HeLa cells (PMN + HeLa), as determined by RNAseq analysis. Color code is reported at the bottom. **c** Violin plot (SPSS Statistics) of gene expression patterns reporting RvD1 up- (yellow) or down-regulated (blue) genes in PMN with a fold-change cut-off of 1.5 (corresponding to 0.58 in the log_2_ scale reported in the figure) as compared to vehicle-treated PMN. PMN: 241 transcripts up- and 6018 down-regulated by RvD1. PMN + HeLa: 74 up, 2813 down-regulated by RvD1. **d** RvD1-regulated genes and significantly associated biological functions in PMN (IPA analysis). Adhesion of neutrophils *p*-value 4.99E-04, activation z-score − 3.057. Homing of PMN p-value 9.64E-03, activation z-score − 5.222. Chemotaxis of PMN p-value 1.45E-02, activation z-score − 5.222. Green symbols: down-regulated genes; red symbols: up-regulated genes. Blue dotted lines: expression leading to inhibition; yellow dotted lines: expression leading to activation; grey dotted lines: expression leading to unpredictable effect of function. Blue boxes: inhibited functions. **e** RvD1-regulated genes and biological functions related to cancer in PMN during co-incubations with HeLa cells (IPA analysis). Invasion of tumor, p-value 3.94E-02, activation z-score − 1.983. Growth of tumor, p-value 2.57E-02, activation z-score − 1.877. See above for symbol codes. **f** Relative HeLa cell growth during co-incubations with vehicle- or 100 nM RvD1-treated blood-derived PMN, as determined by crystal violet staining after 18 h of coincubation. HeLa cells treated with vehicle or RvD1 w/o PMN are shown for comparison. Data are expressed as fold over HeLa cells treated with vehicle *n* = 12. *, *P* < 0.05 (Mann Whitney test). **g** Relative growth of HeLa cells during co-incubations with freshly isolated blood-derived human PMN treated with vehicle or RvD1 (100 nM) and analyzed with impedance-based real time cell analysis (ACEA). Data are expressed as relative HeLa cell growth normalized at the time of addition of vehicle- or RvD1-treated PMN. *n* = 3. ****, *P* < 0.0001 (Wilcoxon matched-pairs signed rank test). **h** Relative growth of human HPV-positive head and neck cells UM-SCC-104, human lung A549 cells, and murine HPV-positive C3 cells, co-incubated with freshly isolated blood-derived human or bone marrow-derived mouse PMN. Data are expressed as fold over cancer cell lines co-incubated with vehicle-treated PMN, indicated in the graph with a dashed line, and determined by crystal violet staining after 18 h of PMN-cancer cells coincubations. *n* = 4 (UM-SCC-104), 8 (A549), and 3 (C3). *, *P* < 0.05; **, *P* < 0.01 (one-sample *t* test)

To uncover molecular signatures of RvD1 bioactions on PMN in HPV-induced cancer, we carried out a genome-wide RNAseq-based analysis of healthy human peripheral blood PMN treated with RvD1 (100 nM) or vehicle and co-cultured with HPV-positive HeLa cells (Fig. 3a). PMN alone were used as control. Transcriptomic analysis showed that RvD1 modified gene expression in both PMN alone and in co-culture with HeLa cells, reducing the levels of 6018 and 2813 PMN transcripts, respectively, while it up-regulated 74 transcripts in PMN alone and 241 in PMN co-cultured with HeLa cells (Fig. 3b-c). Functional analysis carried out with DAVD highlighted that genes modulated by RvD1 (fold change cut-off ±1.5) in PMN were, as expected, primarily involved in immunity, inflammatory response, cytokine and chemokine activity, phagocytosis and chemotaxis. However, RvD1 also regulated genes involved in several less-characterized functions, such as regulation of adaptive immunity and RNA transcription (Supplemental material, Tables S1 and S2). In addition, Ingenuity Pathway Analysis (IPA) revealed that genes modulated by RvD1 were associated with PMN chemotaxis, adhesion and homing (Fig. 3d), confirming that RvD1 limits PMN infiltration into inflammatory sites as previously reported [30]. Notably, in PMN co-cultured with HeLa cells, RvD1 inhibited PMN pathways that hamper tumor growth and invasion (Fig. 3e), suggesting that RvD1 stimulates anti-cancer activities in PMN within a tumor environment.

To test this hypothesis, we exposed blood-derived healthy PMN to RvD1 or vehicle and incubated them with HeLa cells to analyze their impact on cancer cell growth. As shown in Fig. 3f, RvD1 reduced the number of adherent tumor cells co-cultured with PMN, while it had no effect on HeLa cells alone. In addition, a real-time cell growth assay confirmed that RvD1-treated PMN inhibited HeLa cell growth as early as after 5 h and up to 18 h of co-culture (Fig. 3g). Similar results were observed after incubation of human PMN with another HPV-positive cell line (UM-SCC-104) and with the lung epithelial carcinoma cell line A549 (Fig. 3h). RvD1 also prompted purified bone marrow-derived mouse PMN to inhibit the growth of C3 cells, a mouse HPV-positive cell line, thus excluding a cell- or species- specific bias (Fig. 3h). These effects were not due to changes in PMN viability, since the percentage of early and late apoptotic and necrotic PMN was not influenced by RvD1 (Supplemental material, Fig. S5A). Likewise, NETosis, determined by western blot analysis of histone H3-citrullination and by quantification of extracellular DNA in PMN supernatants, was not modified by RvD1 (Supplemental material, Fig. S5B-C). Thus, RvD1 appears to selectively trigger PMN anti-cancer actions.

### RvD1 stimulates a PMN-dependent recruitment of specific anti-tumor monocytes

**Fig. 4:**
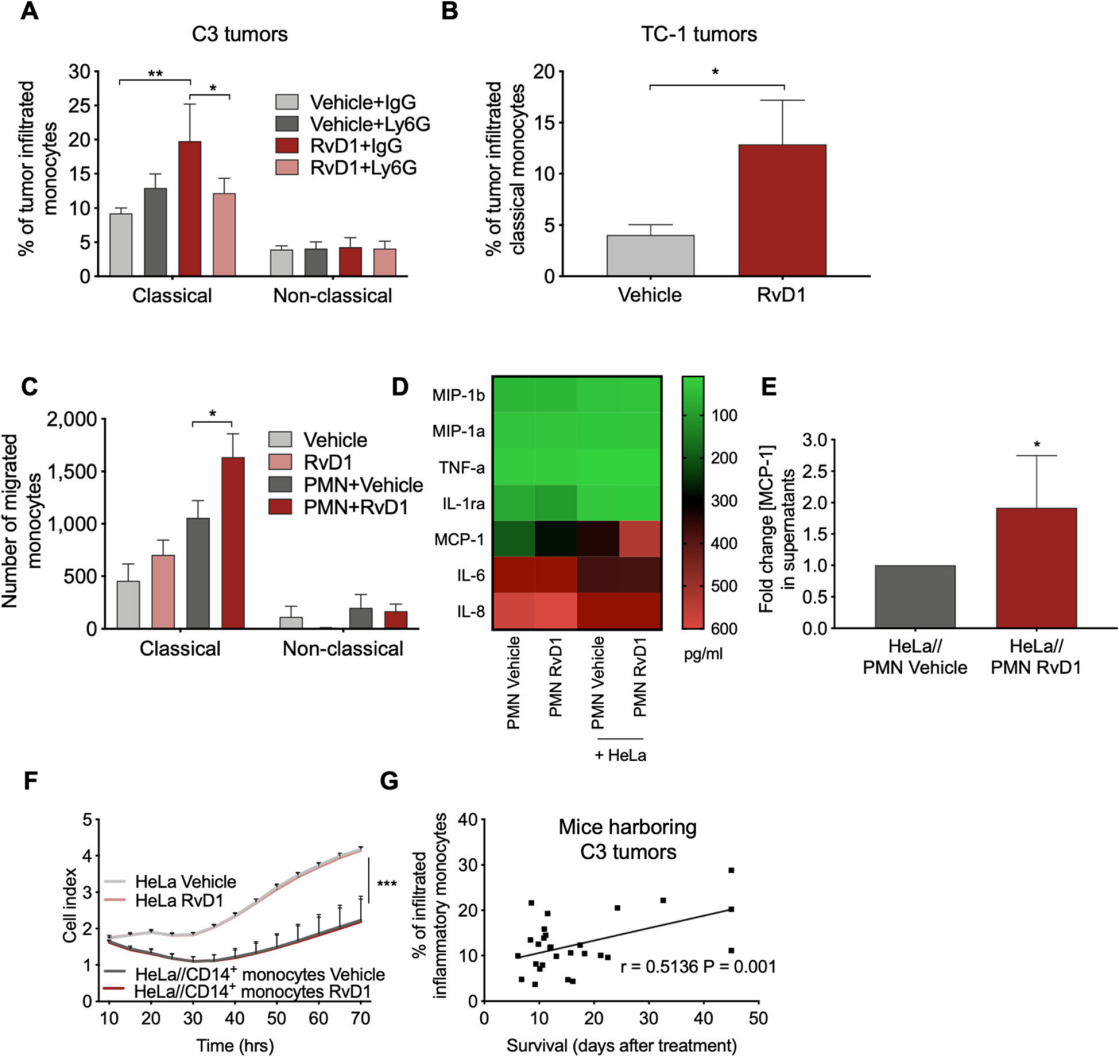
RvD1 prompts a PMN-dependent recruitment of anti-tumor monocytes in cancer models. **a** Percentage of classical (Ly6C^high^) and non-classical (Ly6C^low^) infiltrated monocyte subtypes in tumors from C57BL/6 mice subcutaneously transplanted with C3 cells and treated with vehicle or RvD1 (1 μg/kg) plus anti-IgG or anti-Ly6G, as reported in Fig. 3a. Data are expressed as percentage of myeloid cells as determined by FACS analysis using the gating strategy reported in panel (**a**). *n* = 4–6 mice tumors per group. *, *P* < 0.05; **, *P* < 0.01 (two-way ANOVA with Tukey’s multiple comparisons test). **b** Percentage of classical (Ly6C^high^) infiltrated monocyte subtypes in tumors from C57BL/6 mice subcutaneously transplanted with TC-1 cells and treated with vehicle or RvD1(1 μg/kg) plus anti-IgG or anti-Ly6G as reported in Fig. 3a. Data are expressed as percentage of myeloid cells. *n* = 5–9 mice tumors per group. *, *P* < 0.05 (Mann Whitney test). **c** Number of human monocyte subsets transmigrated in the lower chamber of the Transwell insert 16 h after co-incubations with HeLa cells in the presence or not of PMN and treated with vehicle or RvD1 (100 nM). Number of monocytes are determined by measurements of CD14^+^CD16^−^ (classical) and CD14^low^CD16^+^ (non-classical) events recorded in 30 s of FACS acquisition. *n* = 4. *, *P* < 0.05 (two-way ANOVA and Tukey’s multiple comparison test). **d** Heatmap reporting the average concentration (pg/ml) of the indicated proteins in supernatants from PMN or PMN co-cultured with HeLa cells. **e** MCP-1 concentration in supernatants form HeLa/PMN coculture treated with vehicle or RvD1 (100 nM), as determined by ELISA. Data are expressed as fold change in MCP-1 concentration (pg/ml) compared to HeLa/PMN treated with vehicle. *n* = 6. *, *P* < 0.05 (Kolmogorov-Smirnov test). **f** Growth of HeLa cells during co-incubations with purified human CD14^+^monocytes exposed to vehicle or RvD1 (100 nM) and analyzed with an impedance-based real time cell analysis (ACEA). HeLa cells treated with vehicle or RvD1 in the absence of monocytes were used as control. *n* = 4. ***, *P* < 0.001 (HeLa Vehicle vs HeLa/classical monocytes Vehicle; HeLa RvD1 vs HeLa//classical monocytes RvD1; Wilcoxon matched-pairs signed rank test). **g** Correlation between the percentage of intratumor inflammatory monocytes and survival in mice transplanted with C3 cells. *n* = 26 XY pairs. Pearson r rank correlation was applied to analyze association between variables. Scattered plots with linear regression lines, Pearson r correlation coefficient, and two-tailed *p* value are reported

PMN-regulated recruitment of circulating monocytes represents a key step toward resolution of inflammation [31]. Therefore, to characterize whether stimulation of PMN with RvD1 also affects host inflammation and resolution, we analyzed monocyte recruitment in tumors. Two main monocyte subsets have been identified to date: classical (CD14^+^CD16^−^ in humans and Ly6C^high^ in mice) and non-classical monocytes (CD14^low^CD16^+^ in humans and Ly6C^low^ in mice). In addition to surface marker expression, classical monocytes accumulate in inflamed tissues where they have proinflammatory and antimicrobial functions and can differentiate into MΦs or dendritic cells, whereas non-classical monocytes enter the tissues in the absence of inflammation to replenish the tissue-resident MΦs and dendritic cells [32]. Exploiting the FACS gating strategy reported in Fig. S6A, we found that tumors from mice treated with RvD1 + IgG were infiltrated with a higher number of Ly6C^high^ monocytes, as compared to tumors from vehicle+IgG-treated mice (Fig. 4a). Opposite, quantification of other key immune cells involved in anti-tumor responses, revealed that RvD1 did not significantly modify the infiltration of PMN, CD4, CD8 T cells, and MΦs (Supplemental material, Fig. S7A-C). The RvD1-stimulated recruitment of Ly6C^high^ monocytes was neutralized by the anti-Ly6G antibody, suggesting that RvD1 activates a PMN-dependent enrollment of a specific monocyte subset in C3 cell tumors (Fig. 4a). RvD1 also elicited Ly6C^high^ monocytes infiltration in TC-1 cell tumors (Fig. 4b), ruling out a cell-dependent action.

To confirm these findings in human systems, we evaluated monocyte transmigration through cancer cells in the presence of PMN. To this end, blood-derived monocytes from healthy donors were seeded in the upper chamber of a Transwell insert, and co-cultured with vehicle- or RvD1- treated PMN in contact with HeLa cells in the bottom chamber (Supplemental material, Fig. S6B) as chemotactic stimulus. At the end of co-incubations, phenotype and number of migrated monocytes were evaluated by FACS analysis. Consistent with the in vivo results, RvD1-stimulated PMN enhanced the transmigration of classical, but not of non-classical, monocytes (Fig. 4c). To investigate on mechanisms, we analyzed a panel of immune-inflammatory cytokines released by PMN exposed or not to RvD1 and co-cultured with HeLa cells. As shown in Fig. 4d-e, RvD1 selectively upregulated MCP-1 production, a key cytokine involved in monocyte recruitment at inflammatory sites through stimulation of the CCR2 receptor [33]. These results indicate that MCP-1 secretion by RvD1-treated PMN may be crucial for the recruitment of classical monocytes, expressing higher levels of CCR2 as compared to non-classical monocytes [32], in the neoplastic environment.

To obtain a direct evidence of the pathobiological relevance of infiltrating classical monocytes in our setting, purified classical monocytes (Supplemental material, Fig. S6C) were exposed to vehicle or RvD1, and co-cultured with HeLa cells. As shown (Fig. 4f), classical monocytes inhibited HeLa cell proliferation regardless of RvD1 treatment. Moreover, in vivo we observed a direct correlation between levels of intratumoral Ly6C^high^ monocytes and survival of mice harboring C3 tumors (Fig. 4g), suggesting protective effects of classical monocytes also in vivo. Collectively, these results indicate that RvD1 reprograms PMN to recruit classical monocytes as anticancer effectors.

**Fig. 5:**
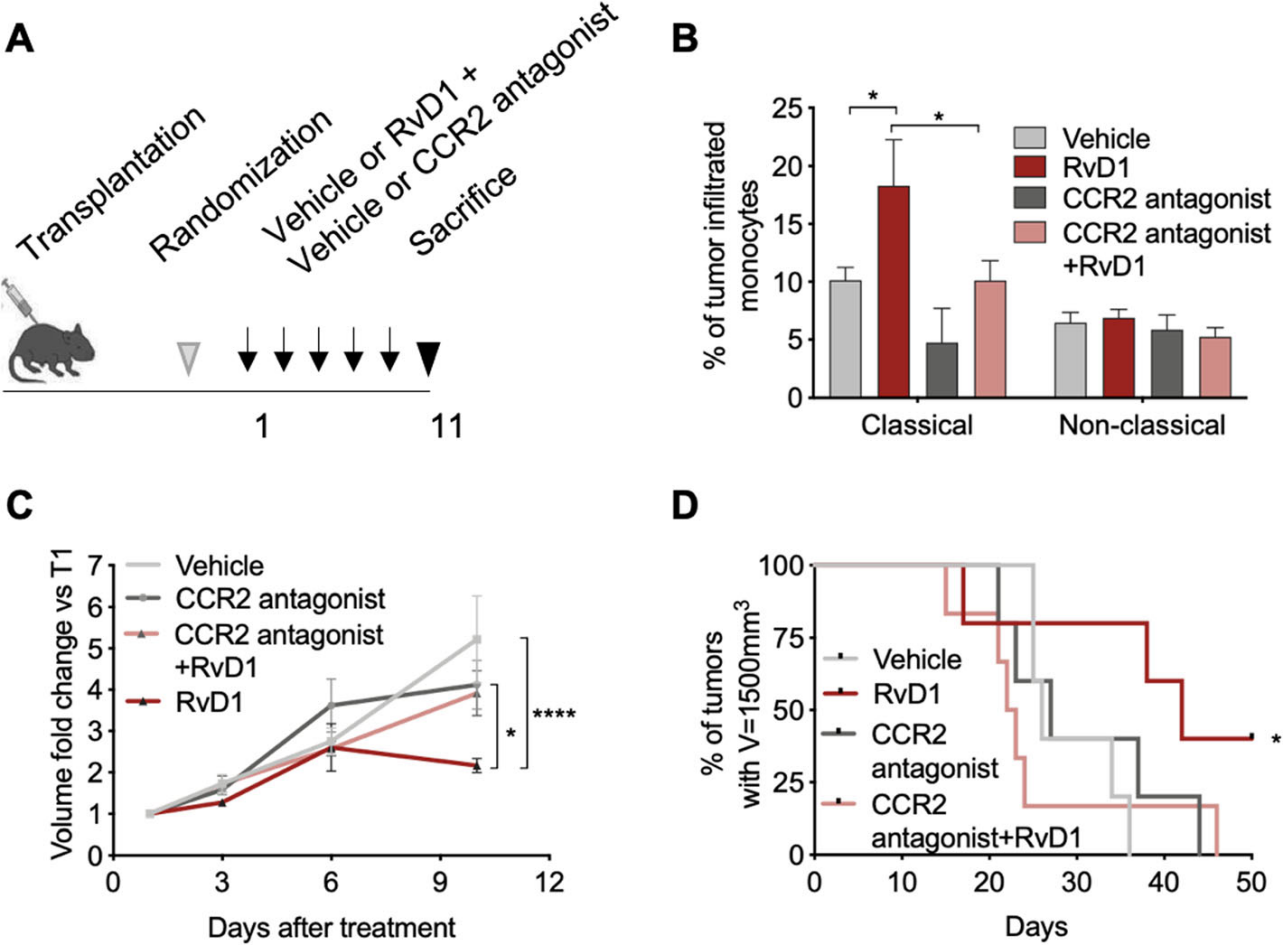
Depletion of tumor-infiltrated Ly6C^high^ monocytes dampens RvD1 anticancer effects. **a** Scheme showing in vivo transplantation of C3 cells in C57BL/6 mice and treatment with vehicle or RvD1 (1 μg/kg) plus vehicle or the CCR2 antagonist SC-202525 (2 mg/kg). **b** Percentage of intratumor classical monocytes as determined by FACS analysis of single-cell dissociated tumors. Data are expressed as percentage of CD45^+^ leukocytes. n = 5–6 mice per group. *, *P* < 0.05 (two-way ANOVA with Tukey’s multiple comparison test). **c** Growth of tumors from C3 cells transplanted in syngeneic mice and treated with vehicle/RvD1 plus vehicle/CCR2 inhibitor. Data are expressed as volume fold change as compared to the initial tumor volume at the start of treatments (T1). *n* = 5–6 mice per group. *, *P* < 0.05 RvD1 vs CCR2 antagonist+RvD1 at day 11. ****, *P* < 0.0001 vehicle vs RvD1 at day 11 (two-way ANOVA and Tukey’s multiple comparison test). **d** Kaplan-Meier curve survival analysis reporting the time for tumors to reach a volume of 1500 mm^3^ (humanized endpoint). *n* = 5–6 mice tumors per group. *, *P* < 0.05 (Log-rank Mantel-Cox test)

To directly corroborate this finding, C57BL/6 mice transplanted with C3 cells and treated i.p. with vehicle or RvD1 were given a CCR2 antagonist that neutralized MCP-1/CCR2 binding and downregulated Ly6C^high^ monocyte infiltration in tumors (Fig. 5a-b). As shown in Fig. 5c-d, the CCR2 antagonist blunted the reduction in tumor growth (mean volume in mm^3^ at day 10: vehicle = 110.4; RvD1 = 68.23, CCR2 antagonist = 88.51; CCR2 antagonist+RvD1 = 155.8) as well as the improvement in survival observed in RvD1-treated mice. Although the CCR2 antagonist may also affect the activity of other immune cells (such as Treg and Th17 [34]) or retains leukocyte subsets in the bone marrow [35], our findings underline the relevance of the MCP-1/CCR2 axis for the RvD1-induced recruitment of monocytes, which are key determinants of RvD1 anticancer effects in vivo.

### Higher monocyte infiltration is associated with a robust immunological response and better prognosis in patients with cervical cancer

**Fig. 6:**
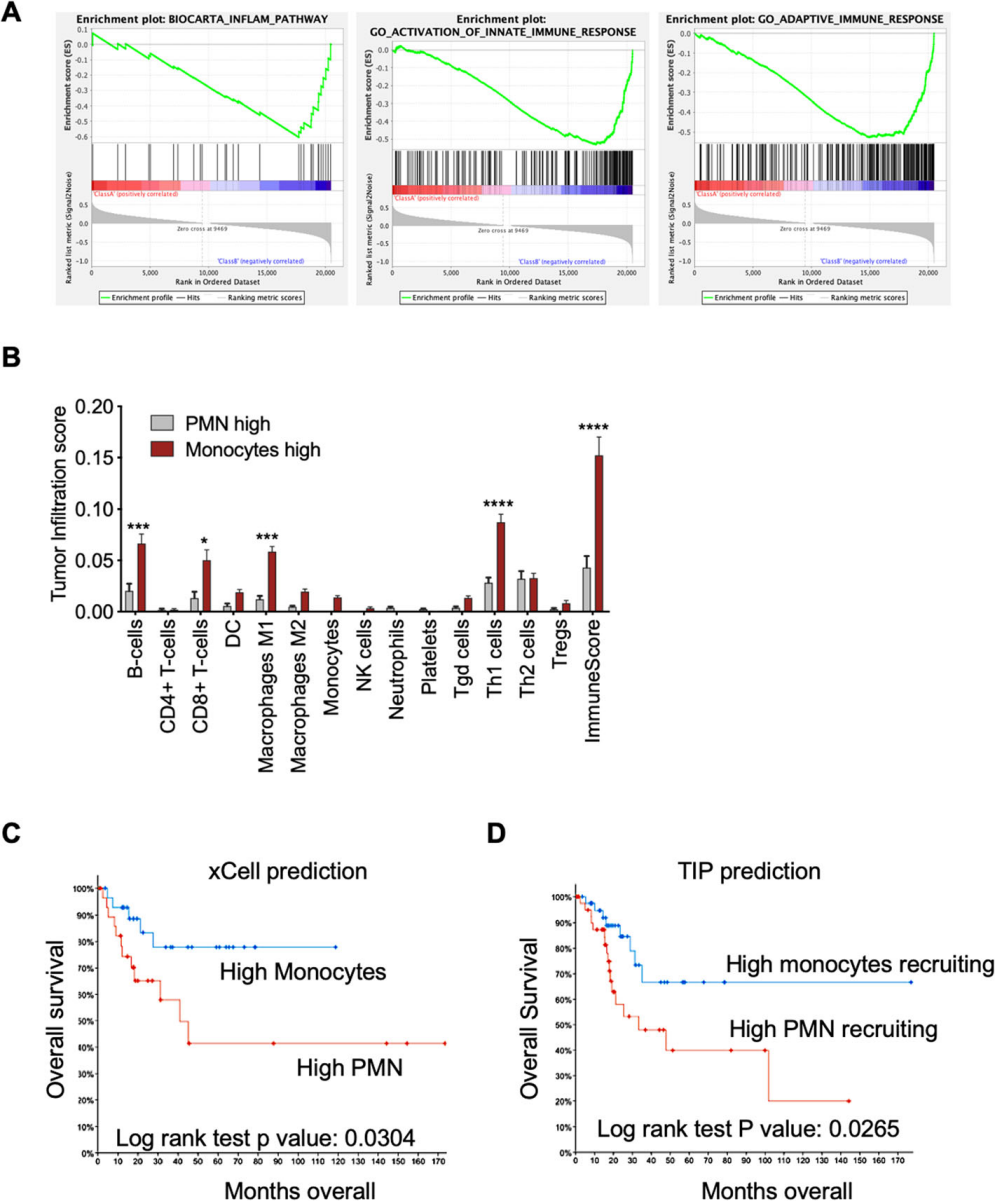
Higher monocyte infiltration predicts stronger immunity and better prognosis in CESC. **a** Gene sets significantly enriched (nominal *P* value < 1%) in CESC patients with higher monocyte infiltration compared to patients with high PMN infiltration, as determined by GSEA. Infiltration levels of immune population were determined by xCell analysis. Patients with the higher monocyte or PMN infiltration levels were selected for the analysis, while samples with overlapping higher monocytes and PMN were excluded. *n* = 41 monocytes high, *n* = 20 PMN high. Gene sets significantly enriched with a nominal *P* value < 1% were considered significant. **b** Tumor infiltration score of key immune cell populations in CESC samples with high monocytes or high PMN, as inferred by xCell. *n* = 41 monocytes high, *n* = 20 PMN high. *, *P* < 0.05; ***, *P* < 0.001; ****, *P* < 0.0001(two-way ANOVA and Sidak’s multiple comparisons test. **c** Kaplan-Meier curve survival analysis of CESC patients stratified as high monocytes (*n* = 41) vs high PMN (*n* = 20) predicted infiltrated cells accordingly to xCell. **d** Kaplan-Meier curve survival analysis of CESC patients stratified as high monocyte recruiting (*n* = 46) vs high PMN recruiting (*n* = 46) accordingly to TIP. Overlapping samples were excluded from the analysis. Clinical data were extracted from the TCGA CESC database and analyzed using the cBioportal software

For a better assessment of the relationship between the immune-inflammatory host response and the clinical outcome and of the clinical significance of our findings, we stratify CESC tumors from TCGA in high PMN vs high infiltrated monocytes. Gene set enrichment analysis (GSEA) of the whole transcriptome of these selected patients evidenced that cancers with high monocyte infiltration were marked by an enrichment in genes involved in inflammatory pathways and innate and adaptive immune responses (Fig. 6a), suggestive of a potent immune-inflammatory reaction against tumor cells. Indeed, characterization of the infiltrating immune cell populations highlighted that tumors with higher monocytes were also greatly infiltrated with key cells for anti-tumor immunity, such as B-cells, CD8 T-cells, M1 MΦs, and Th1 cells, resulting in an elevated ImmuneScore index and higher presence of immune-reactive cells (Fig. 6b). Patients with high monocytes also showed improved prognosis than those with high PMN infiltration, as determined by xCell (Fig. 6c) and TIP (Fig. 6d). Thus, monocyte infiltration is associated with a robust immunological response and better outcomes.

## Discussion

Tumor-associated inflammation is recognized to play pathogenetic roles in cancer [36]. Host inflammatory cells can be detected within the tumor environment, where they engage dynamic interplays with cancer cells, orienting their fate. While a large body of literature has uncovered the central role of tumor associated MΦs [37], less is known regarding PMN, even if their occurrence and function in cancer tissues have been investigated. PMN can either stimulate or inhibit cancer progression, depending on their phenotype or activation state as well as by the tumor stage and anatomic location [4, 8, 11], although the presence of PMN in cancer microenvironments has been predominantly associated with worse clinical outcome [10, 11]. Therefore, despite their lower infiltration levels in tumors as compared to MΦs, their targeting is equally important and may provide an additional or complementary strategy to reduce tumor growth in several malignancies. Along these lines, our bioinformatic analysis with multiple algorithms showed a critical influence of infiltrating PMN on the prognosis of cervical cancer patients (Supplemental material, Fig. S3), suggesting that this type of cancer may be particularly sensitive to PMN bioactions. Despite the implementation of an effective vaccination program, cervical cancer is the second leading cause of death in women aged 20 to 39 years [38]. Experimental and epidemiological evidence indicate an association between chronic inflammation [39, 40] and PMN infiltration [25] with cancer prognosis. In addition, high risk HPV infections are closely related with other malignancies, in particular oropharyngeal cancer, where inflammation and high PMN infiltration negatively correlate with patient survival [41, 42]. Therefore, novel approaches aimed at modulating PMN actions to control HPV-cancers are of wide interest.

Here, using HPV as a model, we tested the hypothesis that reprogramming PMN toward a pro-resolving phenotype may potentiate endogenous anti-tumor host defense mechanisms. In this scenario, pro-resolving mediators may represent ideal molecules, because of their documented capability to regulate, rather than suppress, key actions of immune-inflammatory cells [13]. Anticancer activities of these molecules have been recently reported, although the mechanisms involved remain to be fully elucidated. We found that RvD1 reduces the growth of HPV positive cell-induced tumors in vivo and proliferation of these cells in vitro in the presence of PMN (Figs. 1, 2 and 3); reprograms PMN to acquire a proresolving anticancer phenotype (Figs. 2 and 3); and recruits classical monocytes, which act as key effectors for the inhibition of cancer cell proliferation and tumor progression (Figs. 4 and 5). Thus, despite the identification of PMN subsets in tumors is somehow complex (recently reviewed in [43]), our results suggest that RvD1 may stimulate the formation of antitumoral PMN (N1) that relieves immunosuppression, keeping cancer growth under control. Importantly, our genome-wide RNAseq analysis uncovered a RvD1-induced PMN phenotype compatible with the capability to modulate inflammation and immunity (Supplemental material, Tables S1 and S2). Thus, the immunomodulatory functions of RvD1, largely documented during an acute inflammatory reaction [13], can be exploited to contain the expansion of tumors characterized by a preeminent pathogenetic role of PMN. Importantly, these findings also indicate a more complex action of RvD1 on PMN during an inflammatory reaction that may have important pathophysiological significance. Indeed, early reports showed that RvD1 limit PMN recruitment during inflammation [30], suggesting that PMN clearance is essential to promote resolution. In our model, despite we did not observe any reduction in PMN infiltration in tumors of mice treated with RvD1 (maybe due to the later collection and analysis of tumor-infiltrated leukocytes - i.e. about 12 days after tumor gross appearance, when the cancer-associated chronic inflammation should be fully established), results point to a crucial role for RvD1 in the active reprogramming of PMN actions during inflammation. This substantiates recent evidence designating PMN as key regulators of the initiation of resolution of inflammation and immunity [3, 44].

**Fig. 7:**
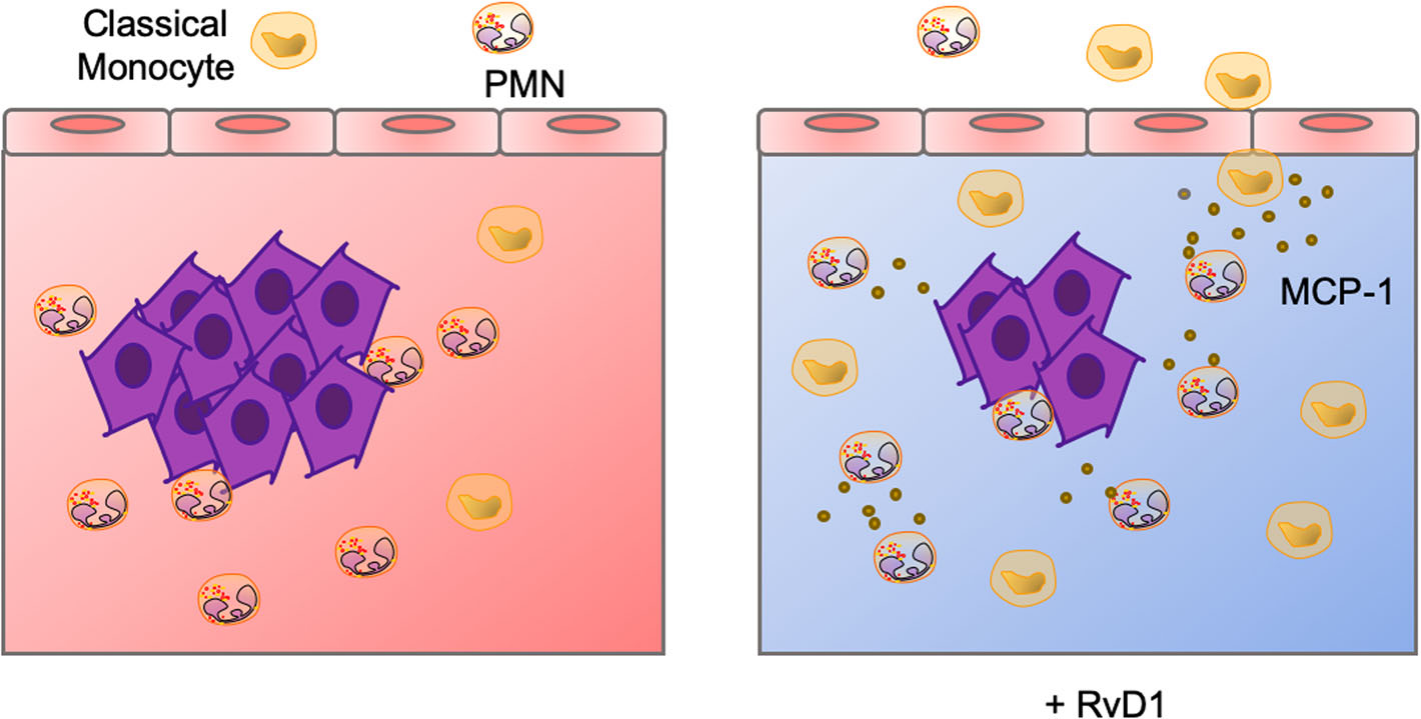
Model of the RvD1-governed PMN-mediated recruitment of anti-cancer monocytes and reduction in tumor growth. In HPV cancers, tumor-infiltrated PMN contribute to fuel cancer growth also by hampering classical monocyte infiltration and by sustaining chronic inflammation. Supplementation of RvD1 activates PMN anticancer activities and stimulates MCP-1 release that, in turn, increases the recruitment of antitumoral monocytes to reduce cancer growth

Supporting this notion, we found that RvD1-stimulated PMN release of higher amounts of MCP-1 to stimulate monocyte chemotaxis. Efficient and timely classical monocyte recruitment is a critical step in acute inflammation and resolution [45]. Similarly to PMN, classical monocytes are reported to have a dual role in cancer, although they have been mainly considered pro-tumoral cells [46]. However, in agreement with early findings [46–48], our present results show that monocytes classically defined as “inflammatory” are able to directly decrease cancer cell growth in vitro and in vivo (Figs. 4 and 5). Importantly, these classical monocytes could differentiate into either dendritic cells or M1 MΦ [32], both involved in anti-tumor immunity, and could also stimulate the adaptive response (reviewed in [47]), thus adding further layers of protection against tumor progression. Therefore, whilst the excessive and uncontrolled monocyte presence in the chronically inflamed tumor milieu sustains inflammation and fuels cancer growth, the timely and selective recruitment of classical monocytes by RvD1 may promote the resolution of cancer-associated inflammation as well as the development of the anti-tumor immune response (Fig. 7). This hypothesis finds support in the exploration of large transcriptomic human datasets from cancer biopsies, an approach that has recently lightened the immune landscape of several malignancies [10, 48], and that revealed that CESC tumors with abundant PMN infiltration display a faint immune response as compared with tumors presenting high monocyte infiltration, where inflammation, innate and adaptive immunity are effective and prognosis more favorable (Fig. 6).

In addition to findings presented here, previous work underlines that SPMs, including Rv, have potent pro-resolving and anti-tumor activities by acting on immune and non-immune cells including NK [15], MΦs [16–18], T cell [19] and cancer cells [18]. Given these pleiotropic effects on multiple cell targets, RvD1 could represent an ideal candidate to boost anti-tumor immunity, also in combination with the available immunotherapeutic drugs. Future studies are however needed to establish the impact of RvD1 on the whole anti-tumor immune response.

## Conclusions

In summary, we present novel anti-cancer properties of RvD1. Our present results, obtained in models of human papillomavirus tumors, uncover a peculiar immunoregulatory mechanism of RvD1 actions that may have potential beneficial effects in other types of cancer characterized by a preeminent dysregulation of the immune inflammatory response.

## Supplementary Information

**Additional file 1: Figure S1.** RvD1 reduces HPV TC-1 tumor growth in syngeneic mice. **Figure S2.** Growth of murine and human HPV-positive cells is not directly affected by RvD1. **Figure S3.** Higher PMN infiltration is associated with poorer prognosis in cervical cancer patients from the TCGA database. **Figure S4.** xCell prediction corroborates the anti-tumor role of CD8 T cells and M1 macrophages in CESC. **Figure S5.** RvD1 does not affect PMN viability during co-incubation with HeLa cells. **Figure S6.** Schemes depicting the identification of mouse leukocyte subsets by FACS analysis, and enrichment of human classical monocytes. **Figure S7.** RvD1 does not modify tumor infiltration levels of CD4 and CD8 T cells, PMN and macrophages. **Table S1.** Functional association of genes and pathways selectively regulated by RvD1 in PMN. **Table S2.** Functional association of genes and pathways selectively regulated by RvD1 in PMN co-cultured with HeLa cells.

## Data Availability

The datasets used and/or analysed during the current study are available from the corresponding author on reasonable request.

## Abbreviations

CESC: Cervical squamous cell carcinoma
GSEA: Gene set enrichment analysis
HPV: Human papilloma virus
IPA: Ingenuity pathway analysis
i.p.: Intraperitoneal
MΦ: Macrophage
MCP-1: Monocyte chemoattractant protein-1
NETs: Neutrophil extracellular traps
PMN: Neutrophils
RvD1: Resolvin D1
SPM: Specialized pro-resolving lipid mediators
TCGA: The Cancer Genome Atlas
