# Supplementary material for: Resolvin D1 reduces cancer growth stimulating a protective neutrophil-dependent recruitment of anti-tumor monocytes
Source: J Exp Clin Cancer Res. 2021 Apr 12;40:129. doi: 10.1186/s13046-021-01937-3 (PMC8040222; doi:10.1186/s13046-021-01937-3)
Supplement: Supplementary file 1 — Additional file 1: Figure S1. RvD1 reduces HPV TC-1 tumor growth in syngeneic mice. Figure S2. Growth of murine and human HPV-positive cells is not directly affected by RvD1. Figure S3. Higher PMN infiltration is associated with poorer prognosis in cervical cancer patients from the TCGA database. Figure S4. xCell prediction corroborates the anti-tumor role of CD8 T cells and M1 macrophages in CESC. Figure S5. RvD1 does not affect PMN viability during co-incubation with HeLa cells. Figure S6. Schemes depicting the identification of mouse leukocyte subsets by FACS analysis, and enrichment of human classical monocytes. Figure S7. RvD1 does not modify tumor infiltration levels of CD4 and CD8 T cells, PMN and macrophages. Table S1. Functional association of genes and pathways selectively regulated by RvD1 in PMN. Table S2. Functional association of genes and pathways selectively regulated by RvD1 in PMN co-cultured with HeLa cells. [file 13046_2021_1937_MOESM1_ESM.docx]

**Supplemental material**

**
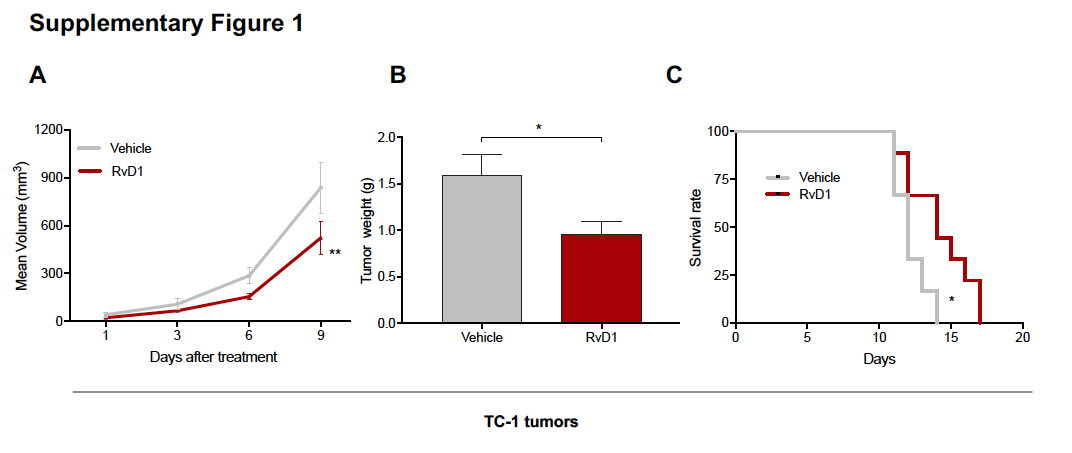
**

**Figure S1. RvD1 reduces HPV TC-1 tumor growth in syngeneic mice.**

**A)** Mean volume of tumors from mice subcutaneously transplanted with HPV-positive TC-1 cells and treated with vehicle or RvD1. Mice were sacrificed 10 days after the initial treatment. *n* = 6-8 mice per group. **, P < 0.01 (multiple t-test with Holm-Sidak method). **B)** Weight of tumors at sacrifice. *n* = 6-8 mice per group *, P < 0.05 (Unpaired t test with Welch’s correction). **C)** Kaplan-Meier curve survival analysis reporting the time for tumors to reach a volume of 1500 mm^3^ (humanized endpoint). *n* = 6-8 mice per group *, P < 0.05 (Log-rank Mantel-Cox test).


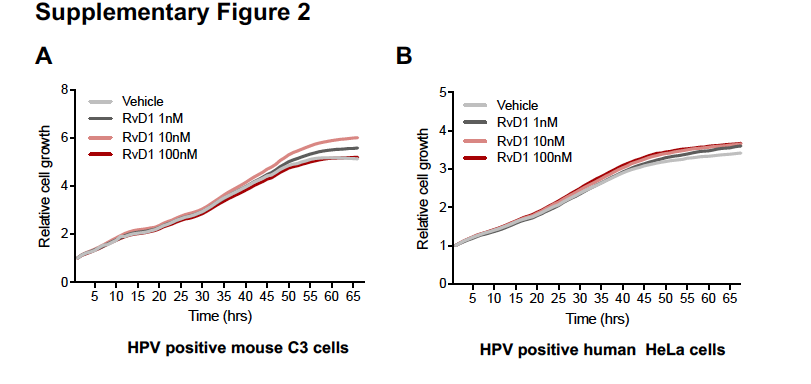


**Figure S2. Growth of murine and human HPV-positive cells is not directly affected by RvD1.**

**A)** Relative cell growth of murine HPV-positive C3 cells exposed to vehicle or RvD1 (0-1-10-100 nM) and analyzed with an impedance-based real-time cell analysis. Data are expressed as relative cell growth normalized at the start of treatments. *n* = 3. **B)** Relative cell growth of human HPV-positive HeLa cells treated with vehicle or RvD1 (0-1-10-100 nM) and analyzed with an impedance-based real-time cell analysis. Data are expressed as relative cell growth normalized at the start of treatments. *n* = 3.

**Supplementary Figure 3**

**A**


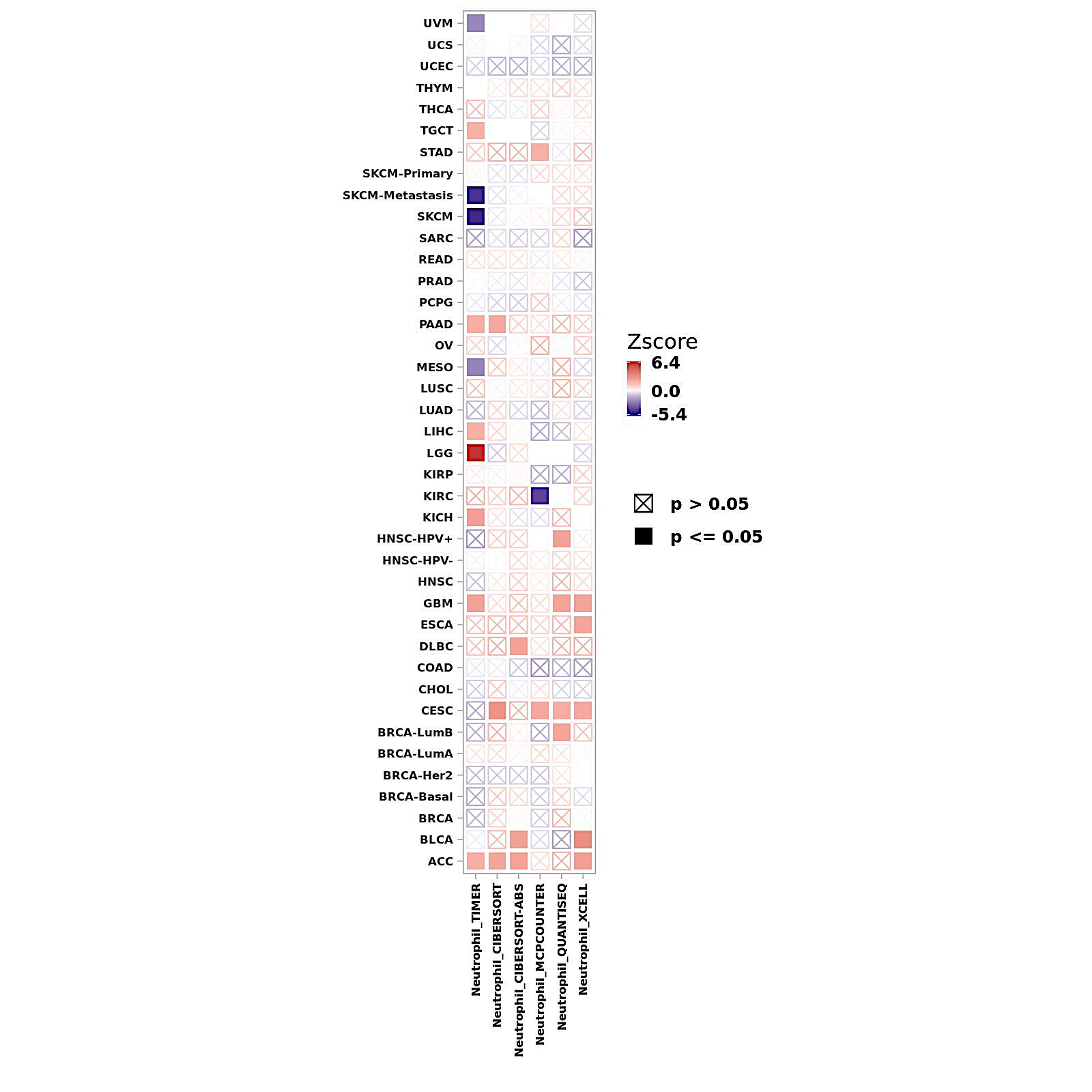

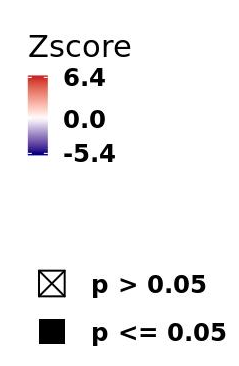


**C**

**D**


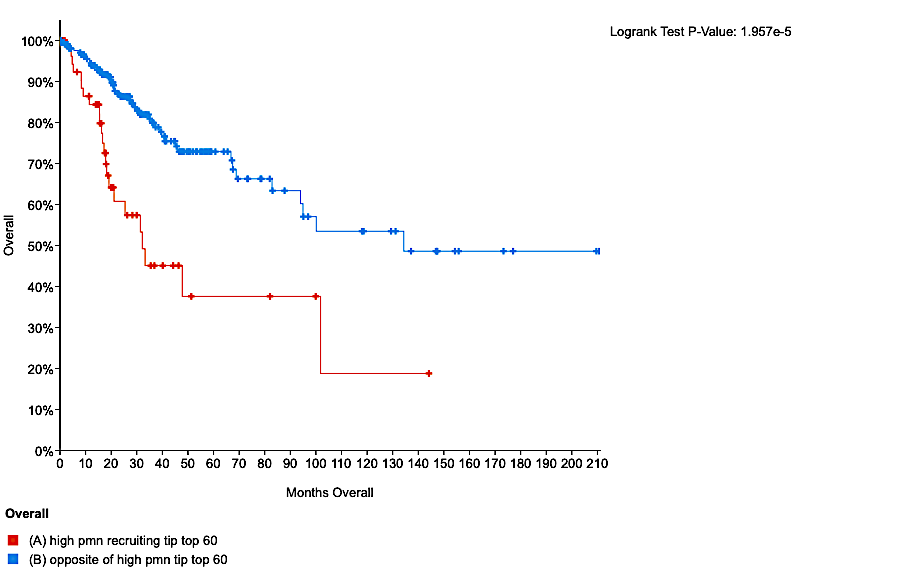


Log rank test P value: 1.957e-5

Months overall

Overall Survival

Low PMN recruiting

High PMN recruiting

TIP prediction


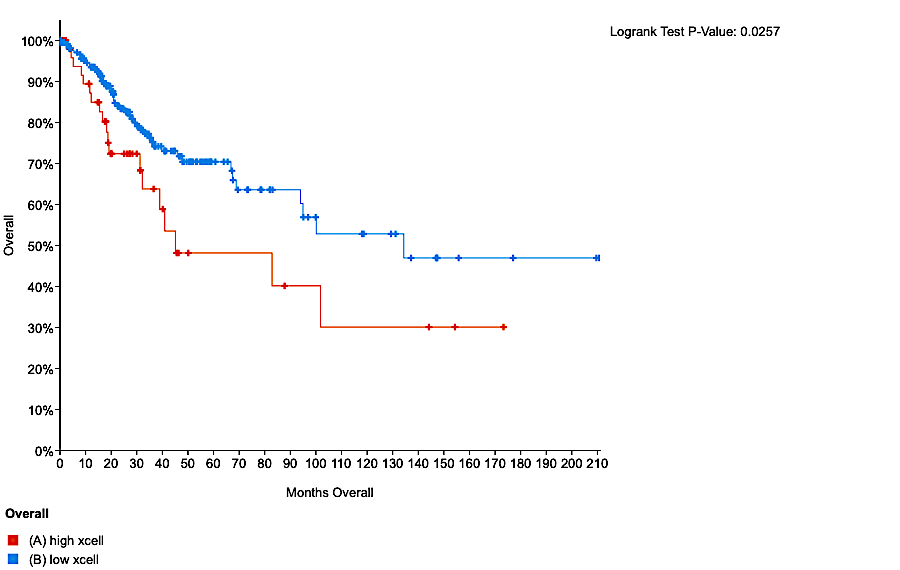


Months overall

Overall Survival

**B**

Low PMN

High PMN

Log rank test P value: 0.0257

xCell prediction

xCell prediction

**E**

**Figure S3. Higher PMN infiltration is associated with poorer prognosis in cervical cancer patients from the TCGA database.**

Predicted infiltrated immune cells levels in TCGA CESC datasets (*n* = 294 patients) accordingly to xCell analysis (9). PMN infiltration is highlighted in black. **B)** Kaplan-Meier survival curve of CESC patients stratified as high (*n* = 55) vs low (*n* = 239) PMN infiltration, as predicted by xCell. Clinical data were extracted from the TCGA CESC database and analyzed using the cBioPortal software (8). Median month overall survival = 45.11 (high PMN group) vs 134.33 (low PMN group). **C)** Predicted PMN infiltration in CESC patients stratified accordingly to the primary tumor TNM stage as recorded in TCGA database. Number of patients’ stage T1A-B: 135; T2A-B: 72; T3A-B: 21; T4: 8. **D)** Kaplan-Meier survival curve CESC patients stratified as high (*n* = 60) vs low (*n* = 237) PMN infiltration as predicted by TIP. **E)** Association between PMN infiltrates and overall survival corrected for age in multiple TCGA datasets using TIMER analysis (10, 11). The heatmap presents the normalized coefficient (Zscore) of association between PMN infiltration and overall survival (red: inverse association; blue: direct association) predicted by different softwares (TIMER, CIBERSORT, MCPCounter, Quantiseq, xCell) in multiple cancer types. Filled squares indicate significant (P < 0.05) associations. TCGA study abbreviations were reported according to the NCI genomic data commons.


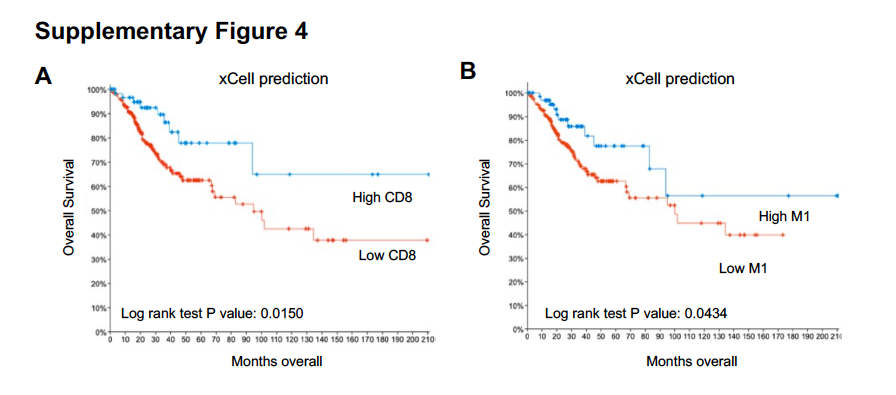


**Figure S4. xCell prediction corroborates the anti-tumor role of CD8 T cells and M1 macrophages in CESC.**

**A)** Kaplan-Meier curve survival analysis of CESC patients stratified as high (*n* = 73) vs low (*n* = 224) CD8 T cell infiltration as predicted by XCell. Clinical data were extracted from the TCGA CESC database and analyzed using the cBioportal software (8); Log-rank test = 0.0150. **B)** Kaplan-Meier survival curve of CESC patients stratified as high (*n* = 73) vs low (*n* = 224) M1 MΦS infiltration as predicted by xCell. Clinical data were extracted from the TCGA CESC database and analyzed using the cBioportal software (8). Log-rank test = 0.0483.

**A**

**B**

**C**

H3- cit

HeLa

PMN RvD1

HeLa

PMN Vehicle


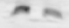

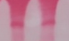


Ponceau

**Supplementary Figure 5**

**Figure S5. RvD1 does not affect PMN viability during co-incubation with HeLa cells.**

**A)** FACS analysis of PMN apoptosis measured by Annexin V/Propidium Iodide (PI) staining. Data are expressed as percentage of early (Annexin V^+^PI^-^), late (Annexin V^+^PI^+^), necrotic (Annexin V^-^PI^+^), or viable (Annexin V^-^PI^-^) PMN exposed to vehicle or RvD1 and co-cultured with HeLa cells for eighteen hours. *n* = 7. PMN kept at 4°C for the same amount of time are reported as negative control of apoptosis. **B)** Representative western blot analysis of citrullinated histone H3 (H3-cit), a marker of NETtosis, released in supernatants by PMN treated with vehicle or RvD1 and co-cultured with HeLa cells for eighteen hours. Supernatants from equal numbers of PMN were loaded. Red ponceau staining is shown as loading control. *n* = 3. **C)** Quantification of extracellular DNA released in supernatants from PMN treated with vehicle or RvD1 and co-cultured with HeLa. *n* = 9. P = ns (one-way ANOVA and Kruskal-Wallis test).


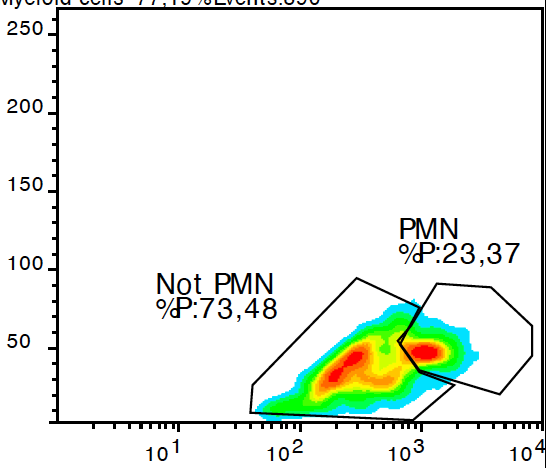


**Supplementary Fig. 6**

**A**

**B**

Monocytes

PMN

HeLa

Transwell insert


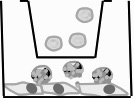


**C**

CD14

SSC


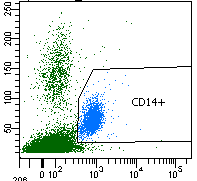

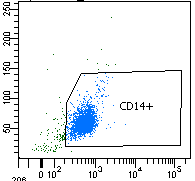


CD14+

CD14+

Pre-enrichment

Post-enrichment

CD45

SSC

CD66a

FSC

CD11b

CD45

F4/80

CD11b

Ly6C

FSC


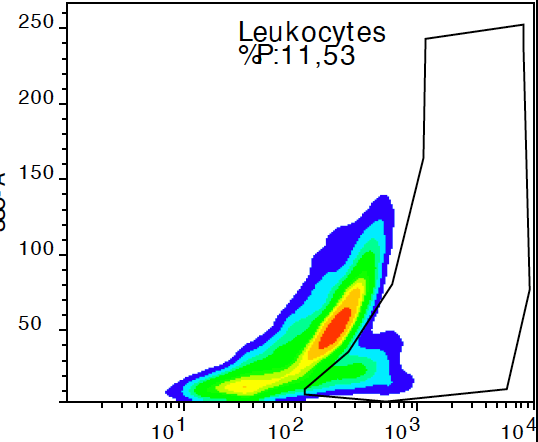

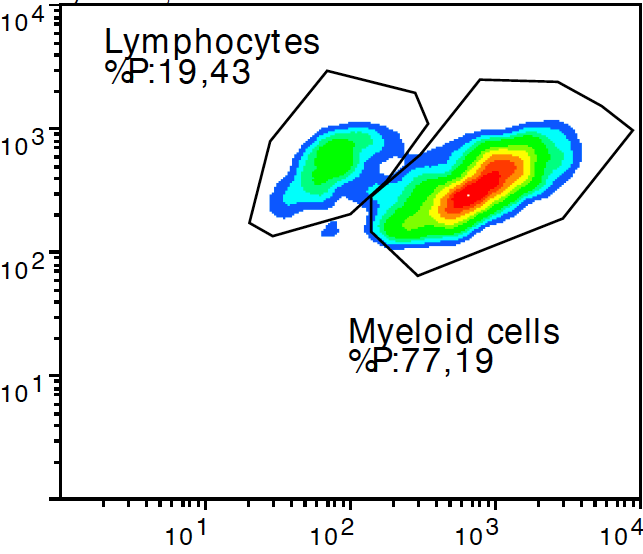

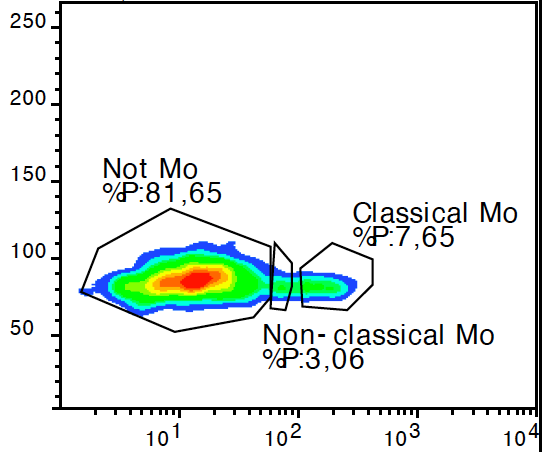

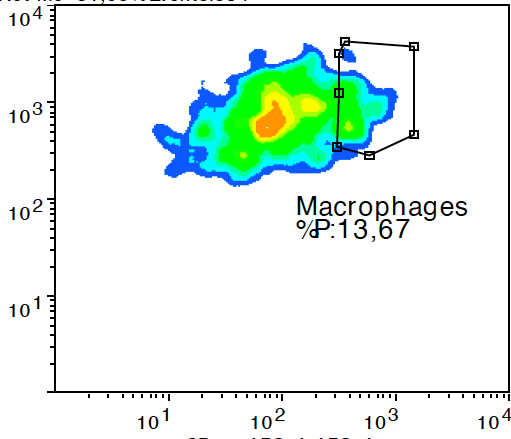

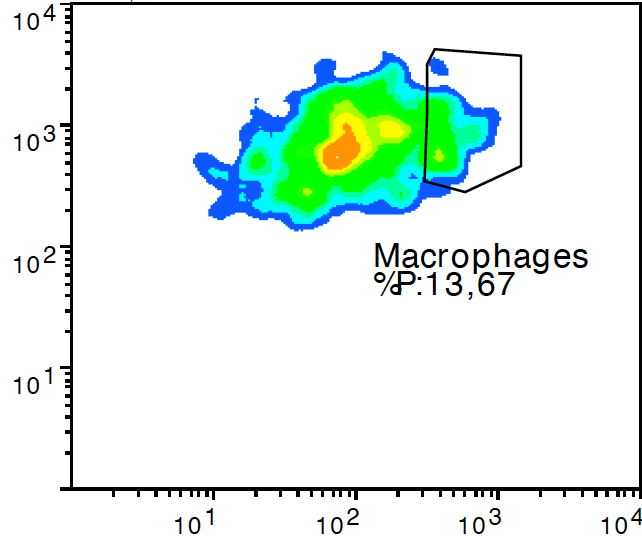


**Figure S6. Schemes depicting the identification of mouse leukocyte subsets by FACS analysis, and enrichment of human classical monocytes**

**A**) FACS gating strategy of monocyte subtypes in tumors from HPV-positive cells (C3 or TC-1) transplanted in the right flank of C57BL/6 mice. Single-cell dissociated tumors were stained with a panel of antibodies to discriminate infiltrated immune cell subpopulations. CD45 positive cells (white blood cells- WBC) are classified as lymphocytes or myeloid cells according to CD11b positivity. PMN are defined as CD45^+^CD11b^+^CD66a^+^ cells. Monocytes (Mo) are identified as CD66a^-^ (not-PMN) Ly6C^+^ cells within the myeloid gate. Ly6C expression allows the identification of non-classical (Ly6^low^) and classical (Ly6C^high^) monocytes. MΦ were identified as F4/80 positive cells in the “not Mo” gate. **B)** Scheme depicting in vitro PMN/cancer cell and monocyte co-incubations to test monocyte migration. PMN and HeLa cells were seeded in the bottom chamber of the Transwell, while monocytes were on the upper chamber separated by a 5 μm pore. **C)** FACS dot plot of blood-derived human PBMCs before and after enrichment of classical monocytes by CD14 MicroBeads (Miltenyi Biotech) and magnetic separation.

**Supplementary Figure 7**

**A**

**B**

**C**

**Figure S7. RvD1 does not modify tumor infiltration levels of CD4 and CD8 T cells, PMN and macrophages.**

**A**) Percentage of tumor infiltrated PMN as determined in single-cell dissociated tumors using the gating strategy reported in panel A. Data are expressed as percentage of CD11b^+^ cells. *n* = 4-7 mice per group. **B)** Percentage of tumor infiltrated CD4 and CD8 T cells in mice subcutaneously transplanted with C3 cells and treated with vehicle or RvD1, respectively, as reported in Figure 1A. Data are expressed as percentage of CD11b^-^ cells. **C)** Percentage of tumor infiltrated MΦ as determined in single-cell dissociated tumors. Data are expressed as percentage of CD11b^+^ cells. *n* = 5-6 mice per group.


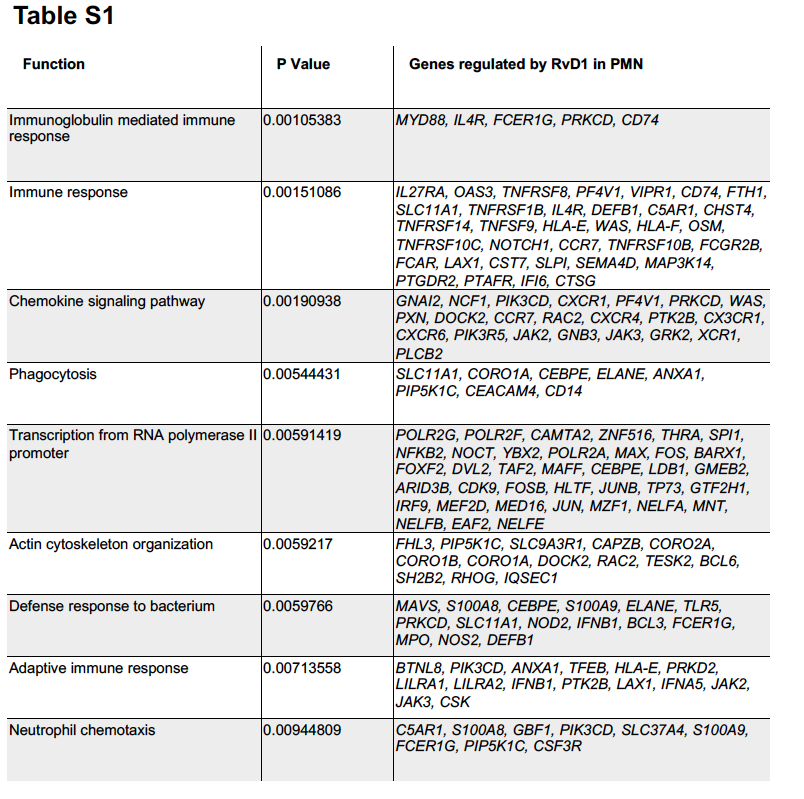


**Table S1. Functional association of genes and pathways selectively regulated by RvD1 in PMN.**

**
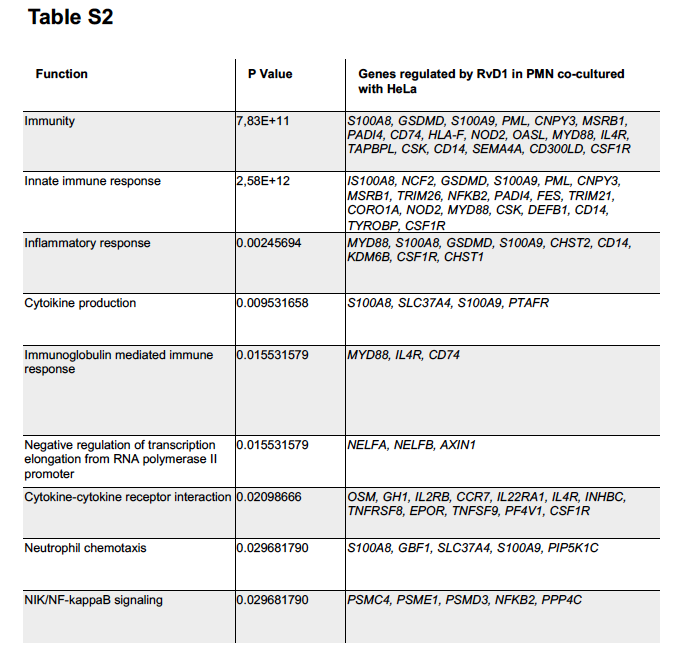
**

**Table S2. Functional association of genes and pathways selectively regulated by RvD1 in PMN co-cultured with HeLa cells.**
